# Genomic diversity of aquaporins across genus *Oryza* provides a rich genetic resource for development of climate resilient rice cultivars

**DOI:** 10.1186/s12870-023-04151-9

**Published:** 2023-03-31

**Authors:** Qasim Raza, Muhammad Abdul Rehman Rashid, Muhammad Waqas, Zulfiqar Ali, Iqrar Ahmad Rana, Sultan Habibullah Khan, Iqrar Ahmad Khan, Rana Muhammad Atif

**Affiliations:** 1 Precision Agriculture and Analytics Lab, Centre for Advanced Studies in Agriculture and Food Security, National Centre in Big Data and Cloud Computing, University of Agriculture Faisalabad, Faisalabad, Pakistan; 2 Department of Bioinformatics and Biotechnology, Government College University Faisalabad, Faisalabad, Pakistan; 3 Department of Plant Breeding and Genetics, University of Agriculture Faisalabad, Faisalabad, Pakistan; 4 Centre for Advanced Studies in Agriculture and Food Security, University of Agriculture Faisalabad, Faisalabad, Pakistan; 5 Centre of Agricultural Biochemistry and Biotechnology, University of Agriculture Faisalabad, Faisalabad, Pakistan; 6 Institute of Horticultural Sciences, University of Agriculture Faisalabad, Faisalabad, Pakistan

**Keywords:** Comparative genomics, Evolution, Haplotype, Major intrinsic proteins, Stress breeding, Water-use efficiency

## Abstract

**Background:**

Plant aquaporins are critical genetic players performing multiple biological functions, especially climate resilience and water-use efficiency. Their genomic diversity across genus *Oryza* is yet to be explored.

**Results:**

This study identified 369 aquaporin-encoding genes from 11 cultivated and wild rice species and further categorized these into four major subfamilies, among which small basic intrinsic proteins are speculated to be ancestral to all land plant aquaporins. Evolutionarily conserved motifs in peptides of aquaporins participate in transmembrane transport of materials and their relatively complex gene structures provide an evolutionary playground for regulation of genome structure and transcription. Duplication and evolution analyses revealed higher genetic conservation among *Oryza* aquaporins and strong purifying selections are assisting in conserving the climate resilience associated functions. Promoter analysis highlighted enrichment of gene upstream regions with cis-acting regulatory elements involved in diverse biological processes, whereas miRNA target site prediction analysis unveiled substantial involvement of osa-miR2102-3p, osa-miR2927 and osa-miR5075 in post-transcriptional regulation of gene expression patterns. Moreover, expression patterns of *japonica* aquaporins were significantly perturbed in response to different treatment levels of six phytohormones and four abiotic stresses, suggesting their multifarious roles in plants survival under stressed environments. Furthermore, superior haplotypes of seven conserved orthologous aquaporins for higher thousand-grain weight are reported from a gold mine of 3,010 sequenced rice pangenomes.

**Conclusions:**

This study unveils the complete genomic atlas of aquaporins across genus *Oryza* and provides a comprehensive genetic resource for genomics-assisted development of climate-resilient rice cultivars.

**Supplementary Information:**

The online version contains supplementary material available at 10.1186/s12870-023-04151-9.

## Background

Plants require light, carbon dioxide, water and nutrients to complete their life cycle. Being sessile, sufficient amounts of water and nutrients are needed to be efficiently absorbed from the surrounding environment through the root system, which continues growing and exploring for the available resources [1]. Water uptake and transport are critically important for maintaining optimum growth and development under an ever-changing abiotic environment. Water transport in the plant body is regulated by three distinct pathways: apoplastic, symplastic and transmembrane pathways. The apoplastic pathway regulates water transport through the xylem, whereas symplastic and transmembrane pathways control cell-to-cell transport of water [2]. The movement of water across cells is governed by aquaporins (AQPs), which are representatives of an ancient major intrinsic proteins (MIPs) superfamily. AQPs are transmembrane proteins that create pores in the membrane of biological cells to facilitate movement of water and other small solutes across the cells [3].

Aquaporins are present in almost all living organisms, except for intracellular bacteria and thermophilic Archaea [1]. AQPs have small molecular weights (26 – 34 kDa) [4] with six transmembrane α-helices, five helix loops and two NPA (asparagine-proline-alanine) motifs [5]. The proteins create homo-tetramer or hetero-tetramer water channel pores in cellular membranes for the permeability of substrate molecules. AQP family has been further classified into five subfamilies based on their intracellular locations and sequence homology. These subfamilies include plasma membrane intrinsic proteins (PIPs), tonoplast intrinsic proteins (TIPs), nodulin 26-like intrinsic proteins (NIPs), small basic intrinsic proteins (SIPs) and uncategorized intrinsic proteins (XIPs) [1]. PIPs with higher molecular weights (~ 30 kDa) constitute the largest subfamily of AQPs, primarily located in plasma membranes for water permeability and further classified into PIP1 and PIP2 subgroups [5]. TIPs with molecular weights in the range of 25 – 28 kDa are most abundant in vacuolar membranes for the transport of water, small solutes and gases, and are further classified into TIP1–5 subgroups. NIPs are predominately located in plasma and intracellular membranes of both leguminous and non-leguminous plants for transport of reactive oxygen species, gases and water, and are further categorized into at least four (NIP1–4) subgroups. SIPs are small, basic but not well-characterized proteins, mostly localized in the plasma membrane and endoplasmic reticulum, participate in water and small solutes transport and are further divided into two (SIP1 and SIP2) subgroups [5]. Just like SIPs, XIPs are primarily located in the plasma membrane and endoplasmic reticulum but are uncharacterized proteins and regulate water, glycerol, hydrogen peroxide and metal transport across biological cells [5, 6].

Aquaporin-encoding genes have been widely studied in diverse crop and plant species including *Arabidopsis* [7], barley [8], cotton [9], maize [10], rice [11, 12], sorghum [13], soybean [14], tomato [15] and wheat [16]. Versatile roles of AQPs in the growth and development of plant species have been reported. These include seed germination, root and leaf growth, hypocotyl and stem elongation, floral development and flower opening, pollen development, anther dehydration, pollen germination, pollen tube elongation, fruit development and ripening, seed dormancy and development and fibre elongation (briefly reviewed in Wang et al. [4]). AQPs also impart detrimental roles in plants survival under abiotic [5, 17–21] and biotic stresses [22, 23]. In the face of rapidly changing climate and depleting crop production resources, identification and characterization of AQPs in the genomes of agronomically important crop are crucial for understanding and exploiting their regulatory roles in developing higher yielding and climate resilient cultivars.

Rice is critical for food and nutritional security. It serves as a staple grain crop and provides a major portion of the daily calorie requirements to half of the world’s population [24]. Rice also serves as a model cereal crop [25]. The genus *Oryza* contains several diploid and tetraploid species which are distributed around the world [26]. The genomes of several cultivated and wild diploid species have been sequenced [27–33], providing an opportunity for comparative genomic studies to get deeper insights into genomics-assisted rice improvement. Genome-wide identification of AQPs has been previously reported in rice [11, 34], however, comparative analysis among cultivated and wild relatives is missing. So far, only a few comparative genome-wide studies have been reported using cultivated and wild rice relatives [35–37]. In this study, a comprehensive genome-wide analysis of aquaporin-encoding genes has been reported across genus *Oryza* along with copy number variations among four important *indica* rice genomes. The identified genes were classified into different subfamilies along with the identification of paralogs and orthologs. Furthermore, gene structures, conserved motifs, gene duplication and evolution, cis-acting elements, micro-RNAs target site prediction and *in-silico* expression analyses were investigated to understand evolutionary patterns, as well as regulatory functions of aquaporins. Moreover, superior haplotypes significantly associated with higher thousand-grain weight were also identified. In conclusion, this research provides a complete genomic atlas of aquaporin-encoding genes across genus *Oryza* for further rice improvement.

## Results

### Identification, classification, and distribution of aquaporin-encoding genes

**Fig. 1:**
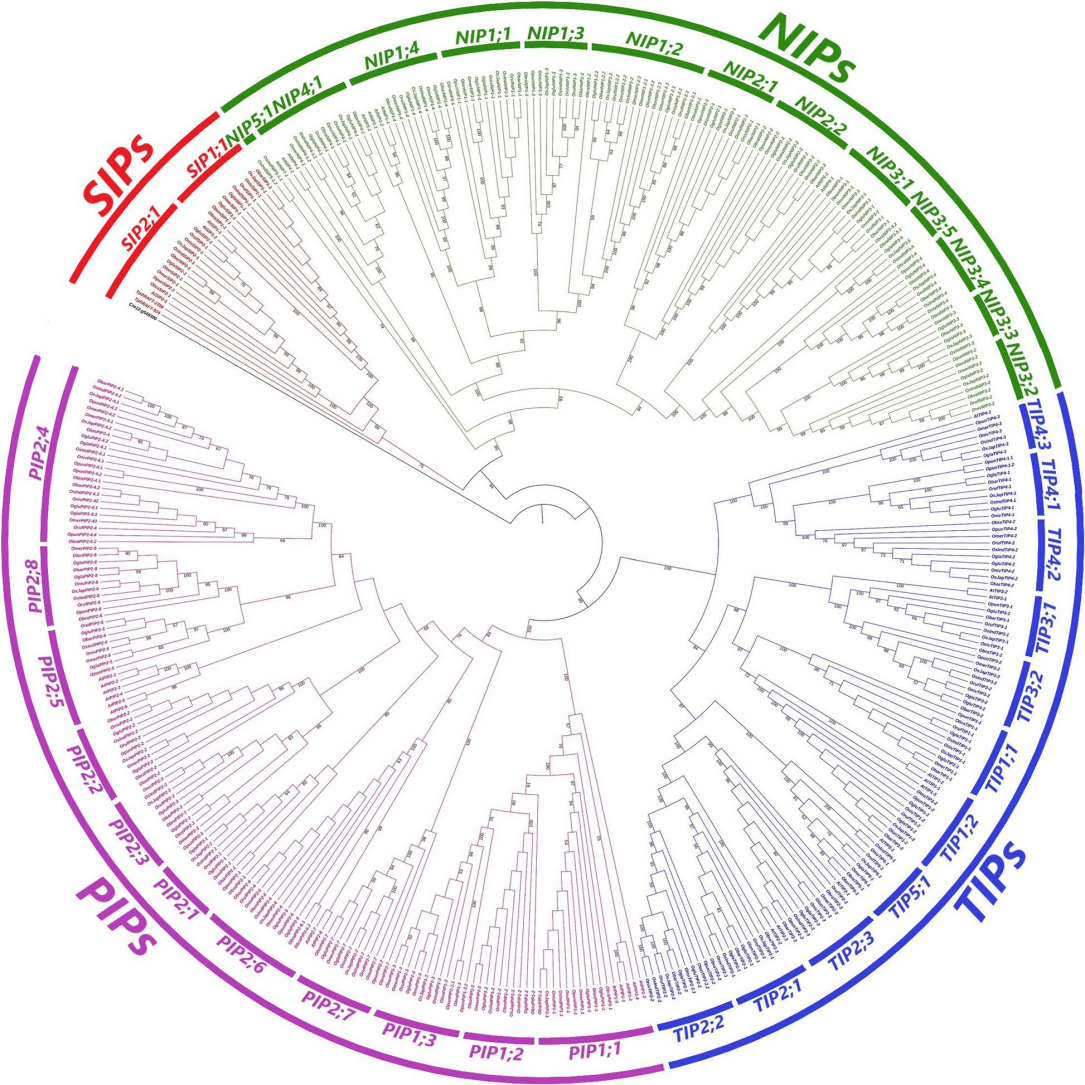
Maximum likelihood comparative phylogenetic tree of plant aquaporins. The ML tree was inferred after multiple sequence alignment of *Arabidopsis*, *Oryza* species, *Chlamydomonas reinhardtii* and *Thalassiosira pseudonana* aquaporin peptide sequences. The two outer bands indicate different aquaporin subfamilies and their isoforms. Bootstrap values equal to and greater than 50% as computed from 1,000 replicates are shown at tree nodes

The query-based gene search approaches using three different keywords yielded a total of 398 aquaporin-encoding genes across 11 rice genomes. After removing redundant and truncated genes, 369 non-redundant and full-length protein-encoding genes were identified and subjected to further analyses. Detailed information on these genes is given in Tables S1 and S2. For classification of genes into different subfamilies, a comparative maximum likelihood phylogenetic tree involving *Arabidopsis* and *Oryza* species genes was inferred. Subfamily names were given based upon orthologous relationships of 35 *Arabidopsis* [7] and 369 *Oryza* genes. All genes were classified into four distinct subfamilies including PIP, TIP, NIP and SIP (Fig. 1). PIP subfamily contained the highest number of genes (130; 35%), followed by NIP (119; 32%), TIP (99; ~ 27%) and SIP (21; ~ 6%) (Table 1), whereas XIP subfamily members were absent in both *Arabidopsis* and *Oryza* genomes. Members of PIP subfamily were further grouped into 11 isoforms (PIP1;1, PIP1;2, PIP1;3, PIP2;1, PIP2;2, PIP2;3, PIP2;4, PIP2;5, PIP2;6, PIP2;7 and PIP2;8), TIP also into 11 isoforms (TIP1;1, TIP1;2, TIP2;1, TIP2;2, TIP2;3, TIP3;1, TIP3;2, TIP4;1, TIP4;2, TIP4;3 and TIP5;1), NIP into 13 isoforms (NIP1;1, NIP1;2, NIP1;3, NIP1;4, NIP2;1, NIP2;2, NIP3;1, NIP3;2, NIP3;3, NIP3;4, NIP3;5, NIP4;1 and NIP5;1) and SIP into two isoforms (SIP1;1 and SIP2;1) (Fig. 1, Table S3). The *O. sativa indica* genome assembly contained highest number of genes (40), followed by *O. nivara* (36), *O. brachyantha* (35), *O. sativa japonica* (35), *O. barthii* (34), *O. glumipatula* (34), *O. punctata* (34), *O. rufipogon* (34), *O. glaberrima* (32) and *O. meridionalis* (29). Whereas the *O. longistaminata* (26) genome harboured least number of genes. Moreover, approximately 52% of the total genes were localized on the 1^st^, 2^nd^ and 4^th^ chromosomes, whereas only a single gene of *O. nivara* localized on the 11^th^ chromosome (Table S1).

**Table 1:** Classification and distribution of aquaporin-encoding genes among *Oryza* genomes

*Oryza* genome	PIPs	TIPs	NIPs	SIPs	Total
*O. barthii*	12	9	11	2	**34**
*O. brachyantha*	12	8	13	2	**35**
*O. glaberrima*	12	10	8	2	**32**
*O. glumipatula*	12	9	11	2	**34**
*O. longistaminata*	9	6	10	1	**26**
*O. meridionalis*	12	6	9	2	**29**
*O. nivara*	12	11	11	2	**36**
*O. punctata*	14	10	8	2	**34**
*O. rufipogon*	11	10	11	2	**34**
*O. sativa indica*	13	10	15	2	**40**
*O. sativa japonica*	11	10	12	2	**35**
**Total**	**130**	**99**	**119**	**21**	**369**

*Chlamydomonas reinhardtii* (*Cre12.g549300*) and *Thalassiosira pseudonana* aquaporin-encoding sequences (*THAPSDRAFT_2356*, *THAPSDRAFT_924*) were also included in comparative tree for determining the ancestral aquaporin subfamily (Fig. 1). Both of the *T. pseudonana* genes were clustered with SIP subfamily members, suggesting that SIP genes are the oldest members of the aquaporins family and all other subfamilies originated from SIP subfamily members. Whereas the single *C. reinhardtii* gene showed a distant orthologous relationship with all other aquaporin subfamilies.

Furthermore, the genomic diversity of AQPs among four *O. sativa indica* genomes was also investigated. The reference genome cultivar 93–11 contained highest number of AQPs (40) followed by Shuhui498 (38), whereas Minghui 63 and Zhenshan 97 both contained 37 AQPs each (Fig. S1A). The comparative analysis highlighted that orthologs of 30 common AQPs were harboured by all four *indica* rice genomes (Fig. S1B). In general, segmental and tandem duplications played evolutionary roles in copy number variations among *indica* genomes (Table S4). The AQPs from reference genome cultivar 93–11 were considered representative of the *indica* group and used in all further analyses.

### Evolutionary conserved motifs and intron–exon distribution among aquaporin subfamilies

**Fig. 2:**
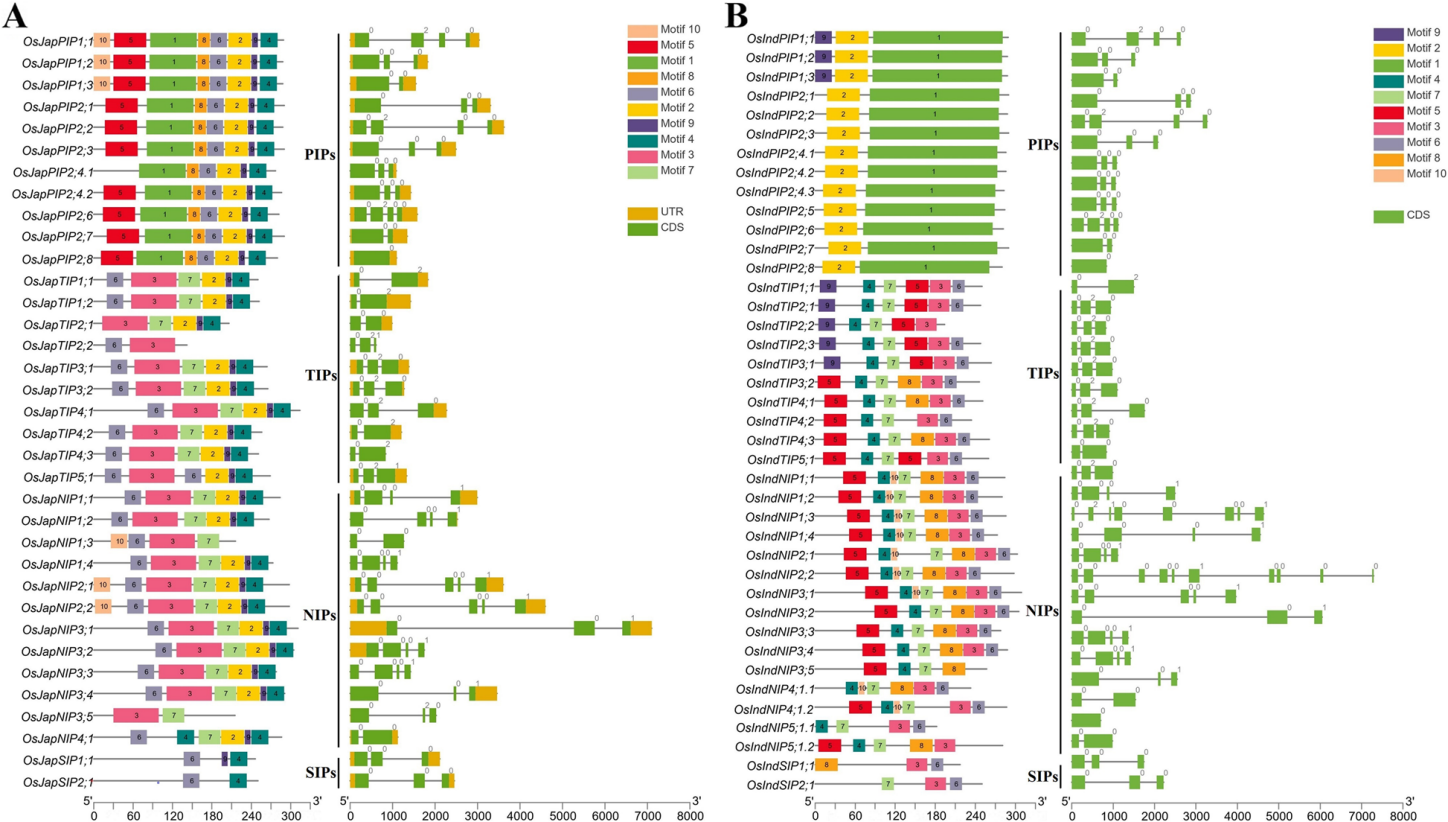
Evolutionary conserved motifs and intron–exon distribution in (**A**) *japonica* and (**B**) *indica* rice aquaporins. Conserved motifs and intron–exon distributions are displayed on the left- and right-hand sides of both figures, respectively. Whereas subfamily names are mentioned in the middle of both figures

To predict evolutionarily conserved motifs among members of different aquaporin subfamilies, the amino acid sequences were subjected to Multiple Em for Motif Elicitation tool. Members of all subfamilies contained at least one MIP domain encoding motif across all *Oryza* genomes (Fig. 2 and Figs. S2–S10). PIP, TIP and NIP subfamilies members harboured 2–8 MIP domain encoding motifs, which were predicted by InterProScan to be associated with transmembrane transport of materials (GO:0,055,085) (Table S5). Similarly, SIP subfamily members contained only 1–3 MIP domain encoding motifs. In general, TIP and NIP members shared conserved motifs of shorter amino acid lengths, whereas PIP members shared motifs of comparatively longer amino acid lengths (Fig. 2 and Figs. S2–S10). Overall, these results support evolutionary phylogenetic relationships among aquaporin subfamilies and suggest an involvement of identified aquaporins in transmembrane transport of materials across rice plant cells.

Gene structures were drawn to explore the intron–exon distribution and structural differences. Majority of aquaporins contained ≥ 2 introns, with some having zero or one intron across all *Oryza* genomes (Fig. 2 and Figs. S2–S10). In general, NIP and PIP members contained relatively complex gene structures (more introns) as compared with the simple structures of TIP and SIP members. Moreover, majority of the introns containing genes had zero intron phases, with several genes having second intron phases, whereas only a few genes with first intron phases. Collectively, these results indicate that relatively complex gene structures of AQPs provide an ideal “evolutionary playground” and serve as repositories of *cis*-elements regulating genome organization and transcription.

### Duplication and evolution of aquaporins

**Fig. 3:**
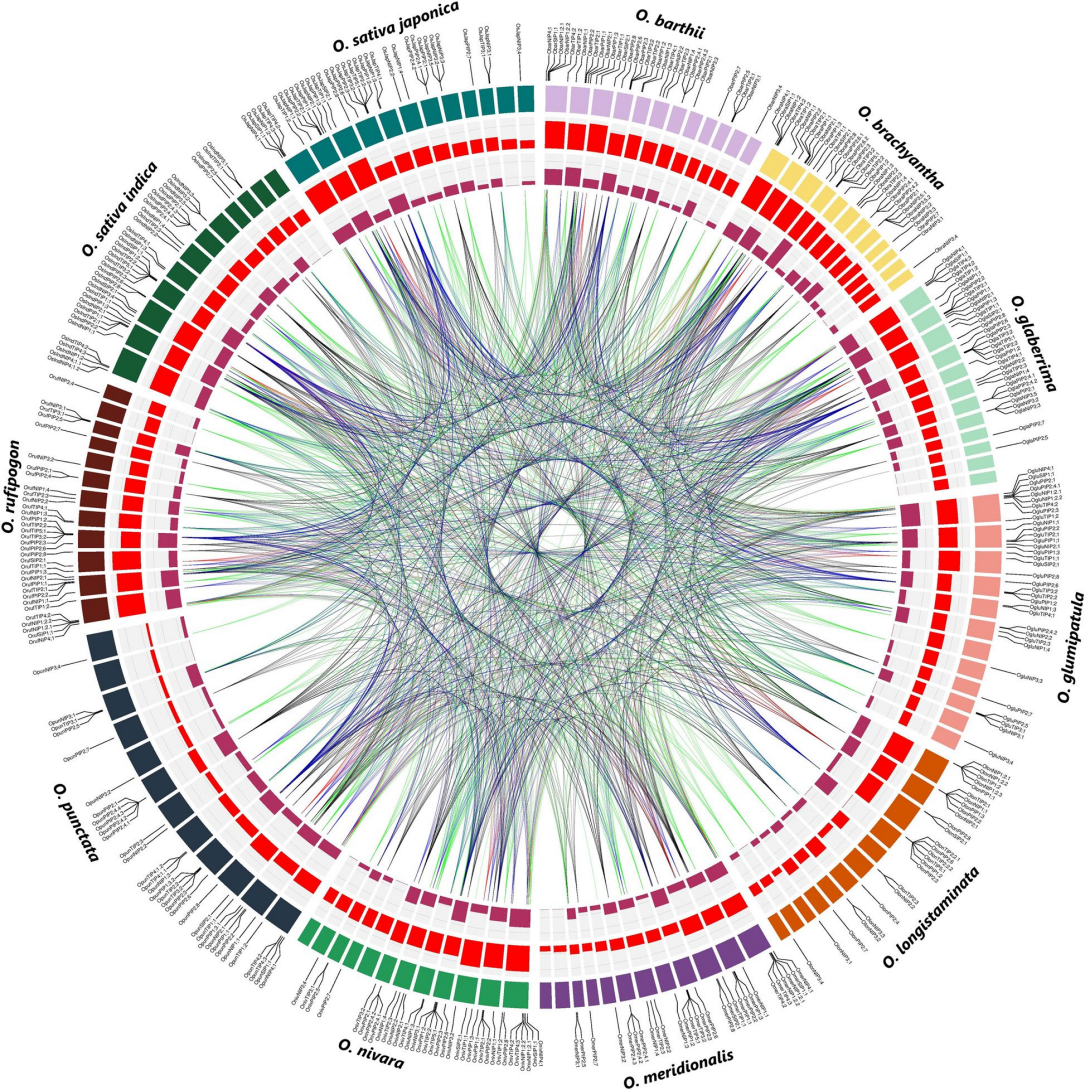
Genomic atlas of aquaporins across genus *Oryza*. A circular diagram from outside to inside is gene names and locations on individual chromosomes, density (count/Mb) of all protein-encoding genes, the density of aquaporin-encoding genes and links indicating duplicated genes among 11 rice genomes

To find duplicated genes, coding sequences were compared against each other and those gene pairs showing ≥ 90% sequence homology were considered duplicated. A total of 1,583 gene pairs corresponding to 351 non-redundant aquaporins of 11 *Oryza* genomes were found to be duplicated (Fig. 3). Of these duplicated gene pairs, at least 117 showed 100% identical protein-encoding sequences among closely related *Oryza* genomes (Table S6). As expected, the duplicated pairs were subfamilies specific and positioned on all rice chromosomes, except for the 11^th^ chromosome of *O. nivara*. Moreover, to gain insights into evolutionary patterns of aquaporin gene family expansion, the coding sequences of duplicated gene pairs were multiple sequence aligned and nucleotide substitution rates were computed. Strong purifying (negative) selections were found to be acting upon approximately 70% of the duplicated gene pairs as their Ka/Ks ratio was < 1, whereas only 2% of the total gene pairs we under positive (Darwinian) selection (Ka/Ks > 1) (Table S6). Furthermore, the duplicated pairs with available Ks information were estimated to be diverged between 0.28 to 70.26 million years ago (MYA). Collectively, these results suggest higher genetic conservation among aquaporins and strong purifying selections operating on codons of cultivated and wild rice aquaporins to conserve their stress-associated functions.

### Regulatory elements in promoter regions of aquaporins

**Fig. 4:**
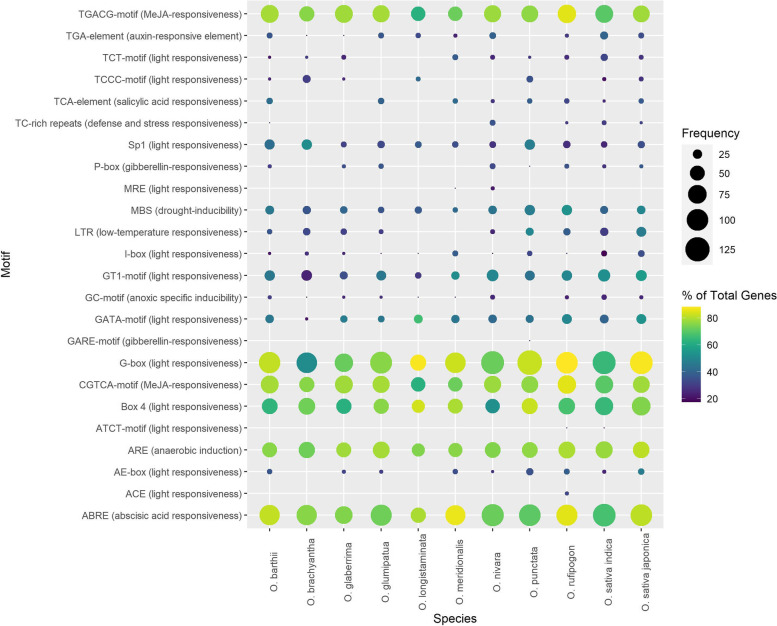
Most frequently occurring cis-acting regulatory elements in promoter regions of *Oryza* species aquaporins

Promoter regions are hotspots for cis-acting regulatory elements (CAREs), which after binding with transcription factors function as transcriptional regulators. In this study, several types of CAREs were predicted in the 2 kbp upstream regions of aquaporins (Table S7). These CAREs are predicted to be involved in diverse biological functions such as phytohormonal signalling, biotic & abiotic stress resistance/tolerance and growth and development-related processes. Notably, the promoters of majority of the aquaporins were extremely enriched with methyl jasmonate, light, abscisic acid and anaerobic induction responsive CAREs across all *Oryza* genomes (Fig. 4). Additionally, the prevalence of drought inducibility and low temperature-responsive CAREs was also noteworthy, although predicted in a smaller number of aquaporins. In general, these results indicate that aquaporins could regulate diverse physiological and biological processes, however, might have highly significant regulatory roles in abiotic stress responses, especially phytohormonal signalling and light responsiveness.

### Micro-RNAs potentially targeting the *O. sativa* aquaporins

**Fig. 5:**
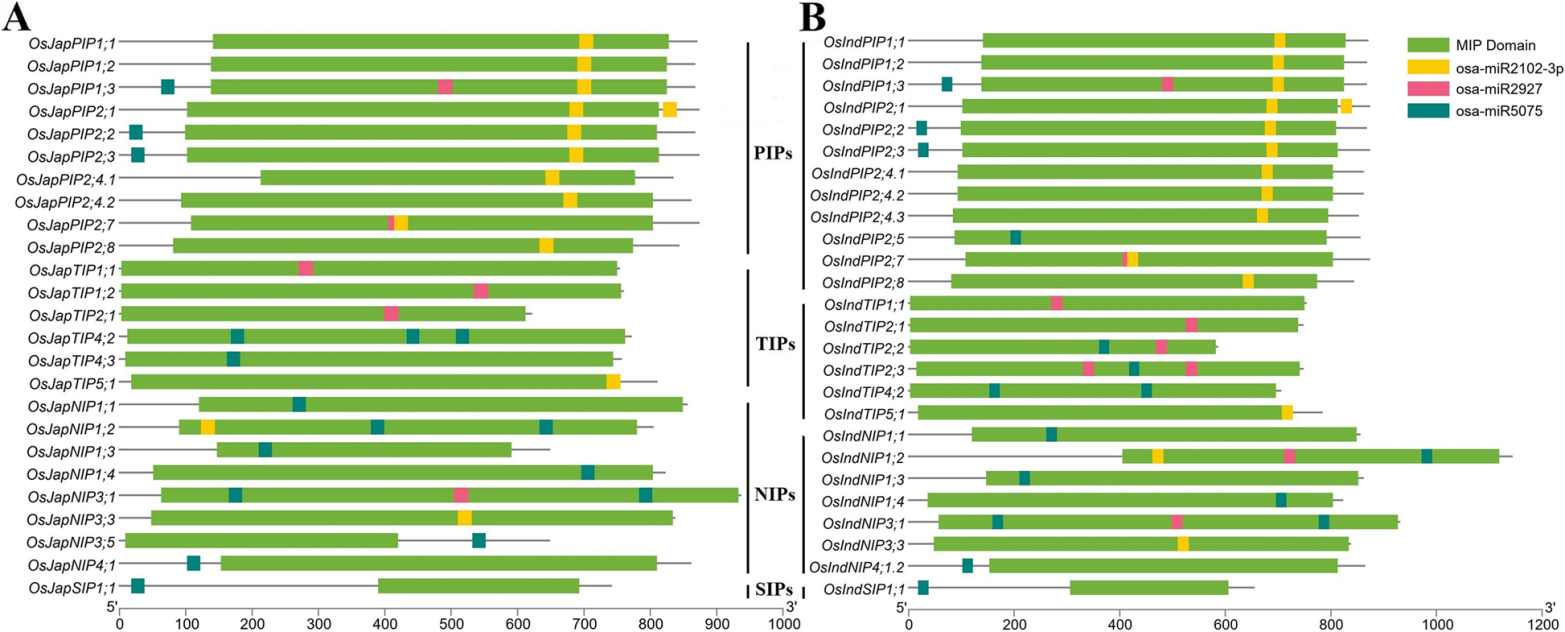
Micro-RNA target sites in (**A**) *japonica* and (**B**) *indica* rice aquaporin transcripts

Micro-RNAs, miRNAs, are reported to post-transcriptionally target the transcription factors and regulate their expression patterns. In this study, several kinds of miRNAs were predicted to potentially target the *O. sativa indica* and *japonica* transcripts (Table S8). Three miRNA types (osa-miR2102-3p, osa-miR2927 and osa-miR5075) were most frequent, as the transcripts of 26 *indica* (65%) and 25 *japonica* (~ 71%) aquaporins contained the putative binding sites of these miRNAs (Fig. 5). Interestingly, osa-miR2102-3p was observed to be potentially targeting the MIP domain sequence of PIP subfamily members of both *O. sativa* sub-species genes. Likewise, osa-miR2927 predominately target the MIP domain in a few gene transcripts of the TIP subfamily, whereas osa-miR5057 potentially targets both TIP and NIP subfamily transcripts of both sub-species. Overall, these results demonstrate that aquaporins are also frequent targets of miRNAs with substantial involvement of osa-miR2102-3p, osa-miR2927 and osa-miR5075 in post-transcriptional regulation of gene expression patterns.

### Expression patterns of aquaporins under phytohormones and abiotic stresses

**Fig. 6:**
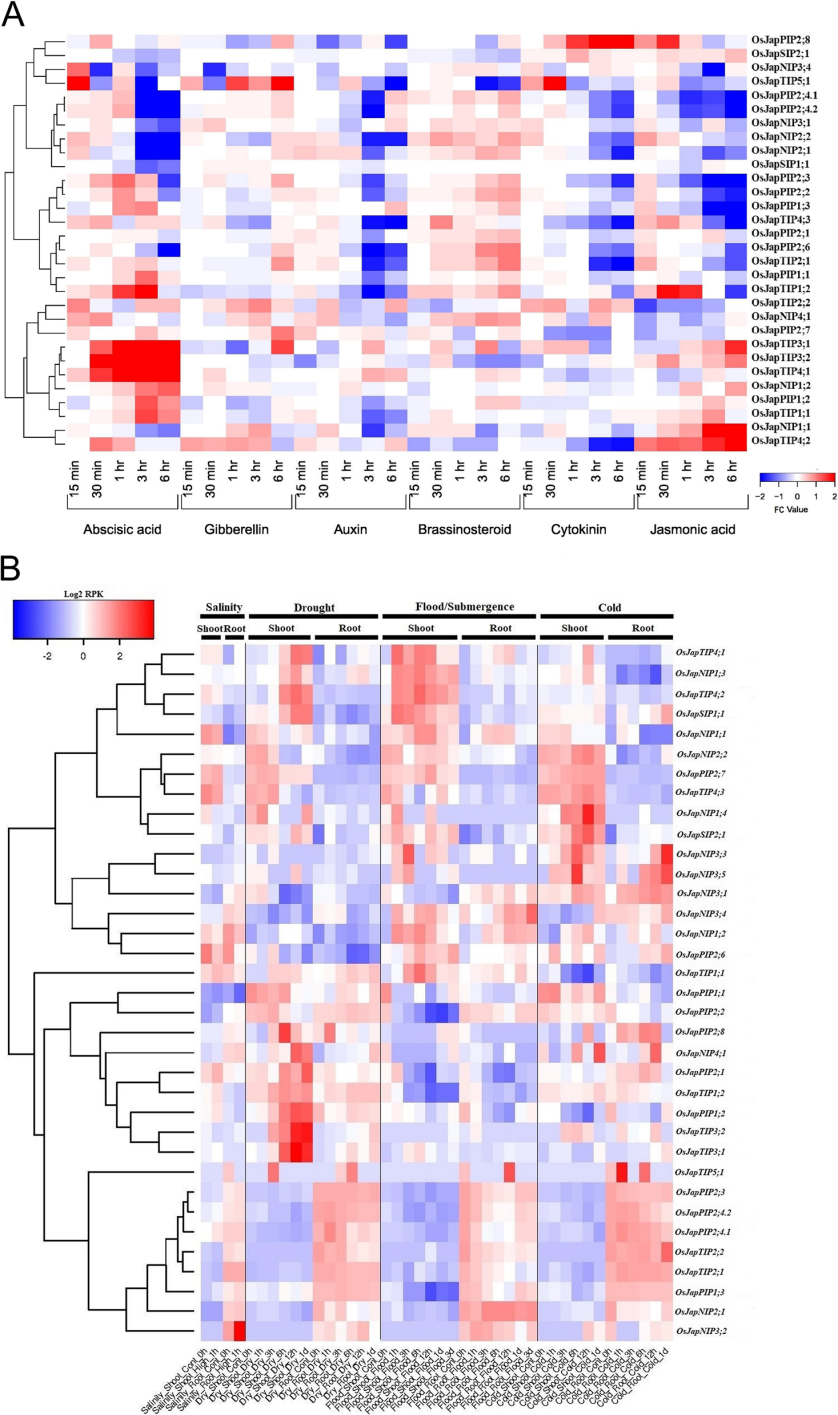
Expression profiles of *japonica* aquaporins in response to (**A**) phytohormone and (**B**) abiotic stress treatments

Since promoters of *Oryza* aquaporins were enriched with phytohormones responsive CAREs, we decided to first examine the global expression patterns in roots of cultivated *japonica* rice under different treatments of six most important plant hormones. In general, after application of phytohormone treatments, expression patterns of the majority of aquaporins were deregulated (Fig. 6A). Nearly all genes showed significant expression perturbations in roots of abscisic acid (ABA) and jasmonic acid (JA) treated rice seedlings, where these genes were ubiquitously up-regulated after ABA and down-regulated after JA treatments, respectively. However, the majority of these genes were significantly down-regulated after three and six hours of auxin as well as cytokinin treatments, whereas marginally up-regulated after brassinosteroid treatments. Notably, three genes of the TIP subfamily (*OsJapTIP3;1*, *OsJapTIP3;2* and *OsJapTIP4;1*) were highly significantly perturbed in response to ABA treatments. Similarly, *OsJapTIP4;2* and *OsJapNIP1;1* were highly significantly up-regulated after JA treatments. Collectively, these global expression patterns indicate important roles of aquaporins in perception, signal transduction and stimulation of defence mechanisms against different stresses in general and phytohormonal stresses in particular.

Moreover, expression patterns in roots and shoots of *japonica* rice under four different abiotic stress conditions were also investigated to explore climate resilience associated functions of aquaporins. Majority of the genes showed stress responsive expression at at least one time point of any of the four stresses (Fig. 6B). Notably, expressions of few aquaporins were significantly induced after three or two independent stresses, indicating their putative roles in climate resilience. For example, expressions of *OsJapTIP4;1* and *OsJapNIP1;3* were increased in shoots after drought, flood and cold treatments. Similarly, expressions of *OsJapTIP4;2* and *OsJapSIP1;1* were induced after drought and flood treatments, whereas *OsJapNIP3;3*, *OsJapNIP3;5*, *OsJapNIP3;1*, *OsJapNIP1;2* and *OsJapPIP2;6* expressions were induced after flood and cold treatments. However, none of the *japonica* aquaporins showed high root or shoot expression in all four stresses. Interestingly, *OsJapTIP3;1*, *OsJapTIP3;2* and *OsJapTIP4;1* expressions were significantly induced in drought stress (Fig. 6B), just like their higher expressions under ABA stress (Fig. 6A). Likewise, consistent with their expressions under JA treatments, *OsJapTIP4;2* and *OsJapNIP1;1* were highly induced in drought and flood conditions, respectively. These genes along with those which significantly induced under three or two independent stresses could be important targets for abiotic stress tolerance. Overall, these expression patterns under phytohormones and abiotic stresses suggest putative roles of aquaporins in climate resilience against multiple abiotic stresses.

### Significant haplotypes for higher thousand grain weight

**Fig. 7:**
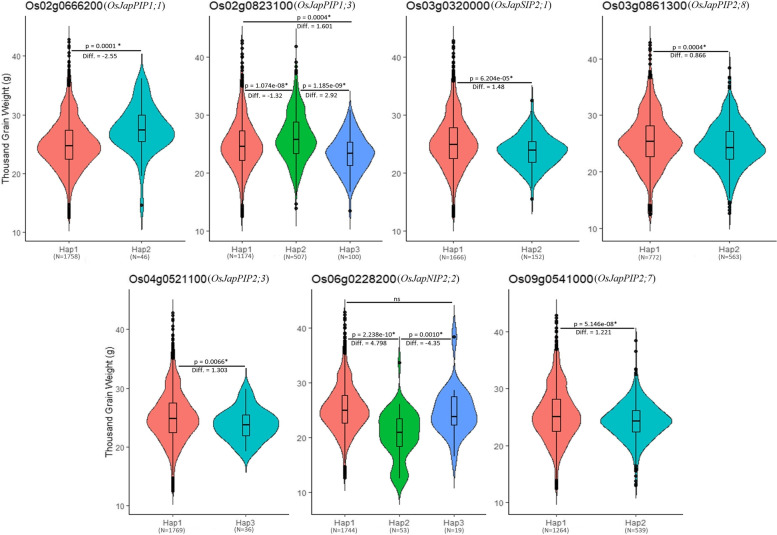
Significant haplotypes in *japonica* rice for higher thousand-grain weight

Keeping in view the paramount roles of aquaporins in abiotic stress tolerance and its impact on rice grain yield, we choose to take advantage of recently reported rice pangenome data and mined significant haplotypes associated with thousand-grain weight (TGW). For this purpose, genome variation and phenotypic data of seven *O. sativa japonica* aquaporins having orthologs in all studied rice genomes were comprehensively examined. A total of 16 haplotypes (two for *OsJapPIP1;1*, *OsJapPIP2;3*, *OsJapPIP2;7*, *OsJapPIP2;8* and *OsJapSIP2;1* each and three for *OsJapPIP1;3* and *OsJapNIP2;2* each) with significant variations in their TGW data were identified (Fig. 7, Table S9). For *OsJapPIP1;1* and *OsJapPIP1;3* haplotypes 2 (Hap2) were found to be superior, as rice accession harbouring Hap2 possessed higher mean TGW as compared with accessions harbouring Hap1 and/or Hap3. Similarly, Hap1 of *OsJapPIP2;3*, *OsJapPIP2;7*, *OsJapPIP2;8*, *OsJapSIP2;1* and *OsJapNIP2;2* were found to be superior than Hap2 and/or Hap3. In general, superior haplotypes demonstrated a higher thousand-grain weight difference ranging from 0.866 g (*OsJapPIP2;8*) to 4.798 g (*OsJapNIP2;2*) than the ordinary haplotypes. Overall, these results indicate that superior haplotypes for higher TGW are predominately contributed by modern rice cultivars and provide a supreme genetic resource for haplotype-led breeding of higher-yielding rice cultivars.

## Discussion

### Aquaporins are vital for life cycle completion of rice plants

Plants have to efficiently utilize available resources and withstand harsh climatic conditions to complete their life cycle. Aquaporins are important regulatory proteins which mediate diverse biological roles during the growth and development of crop plants including rice. Previously, a plethora of studies have reported the crucial roles of rice aquaporins in governing important functions. For example, *OsPIP2;2* transports hydrogen peroxide into the cytoplasm to enhance rice resistance against pathogens [38]. Its Overexpression significantly enhanced water transport along with drought tolerance responses, however, gene silencing significantly inhibited these responses [39]. Additionally, the gene also effectively controlled electrolyte leakage from rice cells and maintained cell membrane integrity after the application of physiological drought stress. *OsPIP1;3* is implicated in diminishing bacterial leaf blight, as gene silencing significantly alleviated disease susceptibility in transgenic rice lines [40]. This gene also facilitate CO_2_ transport, modulate photosynthesis efficiency, abrogate bacterial leaf blight virulence and increase rice grain yield under small scale field trials [23]. Moreover, ectopic expression of *OsPIP1;3* in tobacco exhibited higher water-use efficiency, root hydraulic conductivity and photosynthesis rates, leading to greater plant biomass and overall growth of transgenic plants than non-transgenic plants [41]. Other recent studies in *japonica* and *glaberrima* rice have also reported dynamic physiological responses of PIPs to enhance hydraulic conductivity, mesophyll conductance and transpiration efficiency under fluctuating meteorological conditions [42, 43]. Similarly, overexpression of *OsPIP2;4* improved plant growth of a *japonica* rice cultivar (Giza178) under normal and drought stress conditions [44]. Likewise, *OsPIP1;2* modulates rice growth and grain yield by enhancing mesophyll carbon dioxide conductance and sucrose transport [45]. *OsPIP2;3* has been reported to enhance drought tolerance in rice as transgenic plants overexpressing this gene demonstrated significant improvement in plant growth, tissue elongation, fresh biomass, and chlorophyll accumulation under osmotic stress conditions. Whereas *OsPIP2;3* knockout lines showed retarded growth along with physiological defects [46]. Furthermore, aquaporins also mediate arsenite transport in plants by interacting with other genetic players [19, 20]. Here, in this study also, the promoters of identified aquaporins were enriched with CARE’s involved in diverse biological processes and significant expression perturbations were observed under phytohormones and abiotic stress treatments. Moreover, superior haplotypes of seven rice aquaporins were significantly associated with thousand-grain weight. Collectively, these results indicate the paramount roles of aquaporins in the life cycle completion of rice plants.

### Genetic diversity of aquaporins across genus *Oryza* provides rich resource for further improvement

Globally, rice production is seriously threatened by climate change, abiotic and biotic stresses [47]. Among abiotic stresses, drought, heat and salinity are more prominent due to their adverse effects on overall productivity. Limited genetic diversity in gene pools of cultivated rice species necessitates the introgression of useful genes from wild relatives into the cultivated species to improve rice productivity and climate resilience [48]. In this study, a galore of 369 aquaporin-encoding genes were identified from 11 cultivated and wild rice genomes (Fig. 1, Table S1). Among them, a total of 262 (71%) belonged to eight wild rice species, whereas 107 (29%) belonged to three cultivated rice species or sub-species (Table 1), indicating that wild species hold wider genetic diversity of aquaporins (Fig. 2 and Figs. S2–S10) and are important genetic resources for further improvement. Selected aquaporins from wild relatives with large phenotypic effects could be transferred into the cultivated rice for improvement of tolerance against single as well as multiple abiotic and/or biotic stresses.

### Small basic intrinsic proteins are probably the ancestral subfamily of plant aquaporins

Plant aquaporins show a complex evolutionary history and phylogenetic analyses could resolve their evolutionary complexities. Among five plant aquaporin subfamilies, PIPs have been reported to be transmitted through horizontal gene transfer from algae to land plants [1]. TIPs possibly emerged from a PIP ancestor during land plants evolution. Similarly, NIPs might also derive through horizontal gene transfer from an ancestral bacterial gene [49]. Whereas the origin of SIPs and XIPs likely trace back to early plant ancestors [1]. In this study, MIP superfamily members from algae (*Chlamydomonas reinhardtii*) and an early marine plant (*Thalassiosira pseudonana*) were also included in the comparative phylogenetic tree to gain insights into the evolutionary origin of different plant aquaporin subfamilies. Interestingly, both sequences of diatom phytoplankton species *T. pseudonana* were clustered with SIP subfamily members of all rice species (Fig. 1). Whereas single algae MIP sequence exhibited a distant orthologous relationship with all other aquaporin subfamilies. Previously, Abascal et al*.* [50] investigated the diversity and evolution of MIPs in living organisms and also concluded that the origin of SIP subfamily traces back to the common ancestor of all plants which is supported by the findings of the current study. Overall, these results suggest that SIPs are probably an ancestral subfamily of plant aquaporins and their origin traces back to the common ancestor of all land plants.

### Rice aquaporins are under strong purifying selections to conserve their evolutionary functions

Gene duplications are considered a true ‘stuff of evolution’ and play a central role in diversification by generating the raw material for adaptive evolution [51]. Different selective pressures including positive, neutral and purifying selections are exerted on codons of duplicated genes during the extensive history of plants evolution. These selective pressures could alter the structure and function of proteins coded by the duplicated genes. In this study, we noticed that coding sequences of at least 117 and 1,583 duplicated aquaporin-encoding gene pairs were 100% and ≥ 90% identical across all studied rice genomes, respectively (Fig. 3, Table S6), suggesting retention of higher genetic conservation during rice plant evolution. Moreover, the codons of nearly 70% of the duplicated gene pairs were under strong purifying selections (Ka/Ks ratio < 1). Recent studies have also reported higher genetic conservation and strong purifying selections in *Brassica rapa* and cotton aquaporins [52, 53], supporting the current results. Moreover, the extreme conservation of stress-inducible *heat shock protein 70* encoding genes by the simultaneous action of purifying selections and gene conversion has also been recently reported [54]. Together, these results indicate that most of the *Oryza* aquaporins are negatively selected during evolution and strong purifying selections are operating on their codons to eliminate deleterious mutations and conserve stress-associated functions.

### Aquaporins act as guardians of plants against multiple stresses

The broader roles of aquaporins are well documented in plant physiology. Despite their multiplicity in crop genomes, the genetic and physiological investigations along with gene expression and functional characterization studies strongly support the notion that aquaporins could exclusively perform multiple functions [1]. For example, their versatile roles in plants survival under abiotic [5, 17–21] and biotic stresses [22, 23], as well as in growth and development [4] are meticulously reported. In this study, the promoter regions of identified aquaporins were enriched with CAREs associated with multiple biological processes including phytohormone responsiveness, abiotic and biotic stress tolerance/resistance and growth and development-related functions (Fig. 4). Furthermore, the global expression profiles of cultivated *japonica* rice aquaporins were significantly perturbed in response to different treatment levels of six plant hormones and four abiotic stresses (Fig. 6A and B). Previously published literature also support these results as the expression of several plant aquaporins is regulated by abscisic acid [55], gibberellins [56], cytokinins & auxins [57], brassinosteroid [58], jasmonic acid [59] and abiotic stresses [5, 17–21]. In general, these results indicate that aquaporins could fulfil multiple functions under diverse stress environments.

### Improvement of thousand grain weight via haplotype-led approach

The 3 K rice genomes project (3 K RGP) [60] provides a gold mine resource for genomics research and breeding for molecular breeders looking to take advantage of rice genomic diversity and phenotypic data [61]. In this study, we mined the 3 K RGP for superior haplotypes of seven orthologous *Oryza* aquaporins significantly associated with higher thousand-grain weight (Fig. 7). The superior haplotypes exhibited substantially higher TGW than ordinary haplotypes and were predominately contributed by the modern rice cultivars. Selection and transfer of these superior haplotypes via a modern haplotype-led approach could greatly facilitate the development of higher-yielding and climate-resilient rice cultivars. However, before harnessing the genetic potential of these superior haplotypes in applied research programs, multi-environment and multi-season evaluation of a large number of 3 K RGP representative genotypes should be carried out. This approach would not only improve precision breeding but could also pave the way toward a new era of designer crop plant development.

### Comparative genomics facilitating the functional genomics studies for further improvement of rice

Comparative genomics is helpful in understanding evolutionary changes among different species, helping identification of unexplored genes and providing basis for further functional genomics studies. In genus *Oryza*, only a few comparative studies involving genome-wide analysis of important gene families and miRNAs have been reported [35–37]. This study, along with previously reported comparative studies, provides a comprehensive resource of stress responsive gene families and miRNAs for their possible exploitation in basic and applied research programs. Moreover, these studies could help in exploring the untapped genomic diversity, structural and functional organization, and evolutionary dynamics among cultivated and wild rice species. Furthermore, identification of conserved orthologous genes and their superior haplotypes provide a basis for improvement of thousand-grain weight via modern haplotype-led approach. Abiotic and biotic stresses pose a great threat to the global crop production as domestication has significantly eroded the genetic diversity from modern cultivars [62, 63]. Crop wild relatives are treasure troves for enhancement of tolerance and resistance against abiotic and biotic stresses, respectively. The untapped genetic diversity of wild rice species mined through comparative genomics could be used to improve climate-resilience of cultivated rice by employing emerging molecular and genetic engineering technologies [62, 63]. In this way, comparative genomics is facilitating the functional genomics studies for exploiting the available genetic resources to further improve the crop plants.

## Conclusion

This study reports a complete set of 369 aquaporin-encoding genes in 11 cultivated and wild rice species through a comprehensive genome-wide screening approach. The identified aquaporins were classified into PIP, TIP, NIP and SIP subfamilies, among which SIP members are found to be probably ancestral to all land plants. Conserved motifs, gene structure, duplication and evolution analyses revealed higher genetic conservation among aquaporins and strong purifying selective pressure assisting in conserving their evolutionary functions. Several regulatory elements involved in diverse biological processes, different miRNAs targeting MIP domain sequence and significant expression perturbations in response to phytohormones and abiotic stress treatments revealed post-transcriptional regulation and multifarious functions of aquaporins under changing environments. Finally, superior haplotypes for higher thousand-grain weight are reported from a treasure trove of 3,010 rice accessions for boosting grain yield potential. Overall, this study provides a comprehensive resource of rice aquaporins that could be exploited for further crop improvement under rapidly changing global environments.

## Methods

### Database search and sequence retrieval

The Pfam [64] and Ensemble Plants [65] databases were electronically mined (accessed on February 21, 2022) using different keywords (PF00230, MIPs, aquaporins) for comprehensive identification of aquaporin family members in the whole genomes of eleven cultivated and wild rice species including *O. barthii*, *O. brachyantha*, *O. glaberrima*, *O. glumipatula*, *O. longistaminata*, *O. meridionalis*, *O. nivara*, *O. punctata*, *O. rufipogon*, *O. sativa indica* (93–11) and *O. sativa japonica*. For the comparative phylogenetic tree, *Arabidopsis* aquaporin peptide sequences were retrieved from The Arabidopsis Information Resource (accessed on February 28, 2020), whereas a single *Chlamydomonas reinhardtii* (*Cre12.g549300*) and two *Thalassiosira pseudonana* MIP sequences (*THAPSDRAFT_2356*, *THAPSDRAFT_924*) were retrieved from the Ensemble Plants database (accessed on February 21, 2022). Redundant *Oryza* sequences were removed, keeping only the longest transcripts of the full-length protein encoding unique genes. Detailed information along with genomic, coding and protein sequences were retrieved from the Ensemble Plants (Table S1). Additionally, the presence of MIP domain in protein sequences was confirmed through NCBI-CDD (Table S2) [66] before proceeding with further analyses.

### Comparative phylogenetic analysis and identification of paralogous and orthologous gene pairs

*Arabidopsis* and *Oryza* species aquaporin peptides were multiple sequence aligned using MAFFT (v7) by choosing L-INS-i algorithm with default parameters [67]. The maximum likelihood (ML) comparative phylogenetic tree was inferred with IQTree [68] by choosing the WAG + F + R5 best-fit substitution model [69] according to the Bayesian information criterion. The consistency of the ML tree was validated by setting an Ultrafast bootstrap value of 1000 [70]. The final phylogenetic tree was visualized with iTOL v6.5.8 [71]. The classification scheme of Johanson et al*.* [7] was adopted to categorize aquaporins into different subfamilies. Based on the findings of comparative phylogenetic analysis, the genes were classified as orthologous and paralogous. All those genes which belonged to the same rice species were designated as paralogous, whereas those which belonged to the different rice species and clustered together in the same subgroups were designated as orthologous (Table S3). *Oryza* species genes were renamed by following the previously reported naming system for *O. sativa japonica* [11, 34] based upon orthologous relationships.

### Copy number variation among *indica* genomes

Genome assemblies along with annotation files of Minghui 63 (MH63), Shuhui498 (R498) and Zhenshan 97 (ZS97) were retrieved from the Rice Genome Hub (accessed on August 14, 2022) and of 93–11 from the Ensemble Plants databases. The previously identified AQPs of 93–11 were used as queries to BlastP (e-value: 1e-5) against MH63, R498 and ZS97 proteins using TBtools [72]. Moreover, their annotation files were thoroughly searched using the keyword ‘aquaporin’ for comprehensive identification of all AQPs in the corresponding genomes. Finally, duplicated AQPs were removed retaining only the primary transcript of unique genes. A comparative table containing information on orthologous and paralogous genes among four *indica* genomes is provided as Table S4.

### Conserved motifs and gene structure analyses

Protein sequences of all *Oryza* species genes were uploaded in MEME v5.4.1 [73] with these parameters: the number of motifs, 10; motifs occurrence, zero or one; motifs width, 10–300. Other parameters were left at their default values. The predicted motifs were drawn onto the full amino acid lengths of aquaporins using the TBtools. Table S5 provides detailed information on the discovered motifs in all *Oryza* species. Finally, the GFF3 files of all *Oryza* species comprising exons and introns information were subjected to TBtools for drawing the gene structures.

### Gene duplication and evolution analysis

Sequence similarities among coding sequences of all *Oryza* species genes were computed using the Sequence Demarcation Tool (SDT v1.2) [74]. Gene pairs sharing ≥ 90% similarity and an E value of < 1e-10 were considered duplicated and kept for further investigation. After that, nucleotide substitution rates of duplicated gene pairs were computed with TBtools by subjecting the coding and protein sequences. Statistics derived from nucleotide substitution rates were used to predict the type of selection operated on codons of duplicated gene pairs and their approximate evolutionary divergence time. The formula T = Ks/2r × 10^–6^ was used to compute divergence time assuming a value of *r* = 6.5 × 10^–9^ substitutions per synonymous site per year [35]. Table S6 contain detailed information on gene duplication and evolution analysis.

### Identification of cis-acting regulatory elements

The 2,000 base pairs upstream sequences of all genes were retrieved from the RSAT homepage (accessed on March 03, 2022) [75]. The sequence was trimmed when a gene was predicted to be located within the promoter region to avoid overlap. The promoter region sequences were uploaded to the PlantCARE database to predict CAREs [76]. CAREs with core transcription initiation functions (CAT/CAAT and TATA boxes), as well as those without any putative role, were discarded before proceeding with the final analysis. Table S7 contains a summary of promoter analysis results. The final promoter analysis figure was created with the ‘*ggplot2*’ package [77] in R software v4.1.2 [78].

### Micro-RNA target site prediction

The transcript sequences of two *O. sativa* subspecies (*indica* and *japonica*) aquaporins were uploaded on the psRNA Target Server [79] to predict micro-RNAs (miRNAs) possibly targeting the identified genes. A strict criterion with a penalty score of less than equal to 2 was adopted for the prediction of miRNA targets. Finally, the most frequently occurring miRNAs along with their target sites were mapped onto the transcripts of genes using the TBtools. Table S8 contains detailed results of miRNA target site analysis.

### Expression analysis

The normalized expression data (log_2_ fold change) of *O. sativa japonica* aquaporins after treatment with six plant hormones (abscisic acid, gibberellin, auxin, brassinosteroid, cytokinin and jasmonic acid) were obtained from the RiceXPro (accessed on June 24, 2022). Briefly, seven days old seedlings were separately exposed to six phytohormones and differentially incubated for five time periods before total RNA extraction from the root tissues. The detailed expression and normalization procedures were explained by Sato et al*.* [80]. Similarly, normalized mRNA sequencing data (log_2_ reads per kilobase) of *japonica* aquaporins under salinity, drought, cold and flood/submergence conditions were obtained from the Transcriptome Encyclopedia Of Rice (https://tenor.dna.affrc.go.jp; accessed on December 22, 2022) [81]. Nipponbare rice seedlings were separately exposed to stress treatments for different time intervals before total RNA isolation from root and shoot tissues. The detailed experimental and transcriptome analysis procedures were explained by Kawahara et al*.* [81].

### Haplotype analysis

The 3,010 rice genomes variation data was mined from the rice functional genomics & breeding database (accessed on April 04, 2022) [61, 82]. Using this data, several haplotypes have been predicted based upon non-synonymous substitutions in the protein-encoding regions of all rice genes. By selecting seven *O. sativa japonica* aquaporins with orthologs in all other *Oryza* genomes, we identified haplotypes significantly associated with thousand-grain weight [61]. The phenotypic data of TGW from 1,847 rice accessions were retrieved from the rice functional genomics & breeding (RFGB; v2.0) database and used for creating violin plots of significantly associated haplotypes with the ‘*ggplot2*’ package in R software v4.1.2. A brief summary of haplotype analysis is provided in Table S9.

## Supplementary Information

**Additional file 1:** **Table S1.** Detailed information of aquaporin-encoding genes in *Oryza* genomes. **Table S2.** Confirmation of MIP domain in *Oryza* aquaporins through NCBI-CDD. **Table S3.** Paralogous and orthologous aquaporins among *Oryza* genomes. **Table S4.** Aquaporins copy number variation among four important *indica* genomes. **Table S5.** Detailed information on the identified motifs in aquaporins of all *Oryza* genomes. **Table S6.** Details of duplication and evolution analyses. **Table S7.** Summary of promoter analysis. **Table S8.** Detailed results of miRNA target site prediction analysis. **Table S9.** Brief summary of aquaporin haplotypes significantly associated with thousand-grain weight in *japonica* rice.

**Additional file 2:** **Fig. S1A.** Comparative phylogenetic tree of four *indica* rice aquaporins. **Fig. S1B**. Comparison of number of aquaporins among four important *indica* rice genotypes. **Fig. S2.** Evolutionary conserved motifs and intron-exon distribution in *O. barthii* aquaporins. **Fig. S3.** Evolutionary conserved motifs and intron-exon distribution in *O. brachyantha* aquaporins. **Fig. S4.** Evolutionary conserved motifs and intron-exon distribution in *O. glaberrima* aquaporins. **Fig. S5.** Evolutionary conserved motifs and intron-exon distribution in *O. glumipatula* aquaporins. **Fig. S6.** Evolutionary conserved motifs and intron-exon distribution in *O. longistaminata* aquaporins. **Fig. S7.** Evolutionary conserved motifs and intron-exon distribution in *O. meridionalis* aquaporins. **Fig. S8.** Evolutionary conserved motifs and intron-exon distribution in *O. nivara* aquaporins. **Fig. S9.** Evolutionary conserved motifs and intron-exon distribution in *O. punctata* aquaporins. **Fig. S10.** Evolutionary conserved motifs and intron-exon distribution in *O. rufipogon* aquaporins.

## Data Availability

The datasets generated and/or analyzed during the current study are available in the following open access repositories. Accession numbers of all datasets are also provided in the supplementary information tables. Ensemble Plants [https://plants.ensembl.org/species.html] Rice Genome Hub [https://rice-genome-hub.southgreen.fr/] RiceXPro [https://ricexpro.dna.affrc.go.jp/] Transcriptome ENcyclopedia Of Rice [https://tenor.dna.affrc.go.jp/] Rice Functional Genomics & Breeding [https://www.rmbreeding.cn/Index/]

## Abbreviations

MIP: Major intrinsic protein
AQP: Aquaporin
PIP: Plasma membrane intrinsic protein
TIP: Tonoplast intrinsic protein
NIP: Nodulin 26-like intrinsic protein
SIP: Small basic intrinsic protein
XIP: Uncategorized intrinsic protein
Dof: DNA binding with one finger
R498: Shuhui498
MH63: Minghui 63
ZS97: Zhenshan 97
ML: Maximum likelihood
MEME: Multiple Em for Motif Elicitation
Ka: Synonymous substitution
Ks: Non-synonymous substitution
MYA: Million year ago
CARE: Cis-acting regulatory element
miRNA: Micro-RNA
TGW: Thousand grain weight; Hap: Haplotype
3 K RGP: 3 K rice genomes project
NCBI-CDD: National Centre in Biotechnology Information – Conserved Domain Database
SDT: Sequence Demarcation Tool
RSAT: Regulatory sequence analysis tool
RFGB: Rice functional genomics & breeding
