# Supplementary material for: Genomic diversity of aquaporins across genus Oryza provides a rich genetic resource for development of climate resilient rice cultivars
Source: BMC Plant Biol. 2023 Mar 31;23:172. doi: 10.1186/s12870-023-04151-9 (PMC10064747; doi:10.1186/s12870-023-04151-9)
Supplement: Supplementary file 2 — Additional file 2: Fig. S1A. Comparative phylogenetic tree of four indica rice aquaporins. Fig. S1B. Comparison of number of aquaporins among four important indica rice genotypes. Fig. S2. Evolutionary conserved motifs and intron-exon distribution in O. barthii aquaporins. Fig. S3. Evolutionary conserved motifs and intron-exon distribution in O. brachyantha aquaporins. Fig. S4. Evolutionary conserved motifs and intron-exon distribution in O. glaberrima aquaporins. Fig. S5. Evolutionary conserved motifs and intron-exon distribution in O. glumipatula aquaporins. Fig. S6. Evolutionary conserved motifs and intron-exon distribution in O. longistaminata aquaporins. Fig. S7. Evolutionary conserved motifs and intron-exon distribution in O. meridionalis aquaporins. Fig. S8. Evolutionary conserved motifs and intron-exon distribution in O. nivara aquaporins. Fig. S9. Evolutionary conserved motifs and intron-exon distribution in O. punctata aquaporins. Fig. S10. Evolutionary conserved motifs and intron-exon distribution in O. rufipogon aquaporins. [file 12870_2023_4151_MOESM2_ESM.docx]

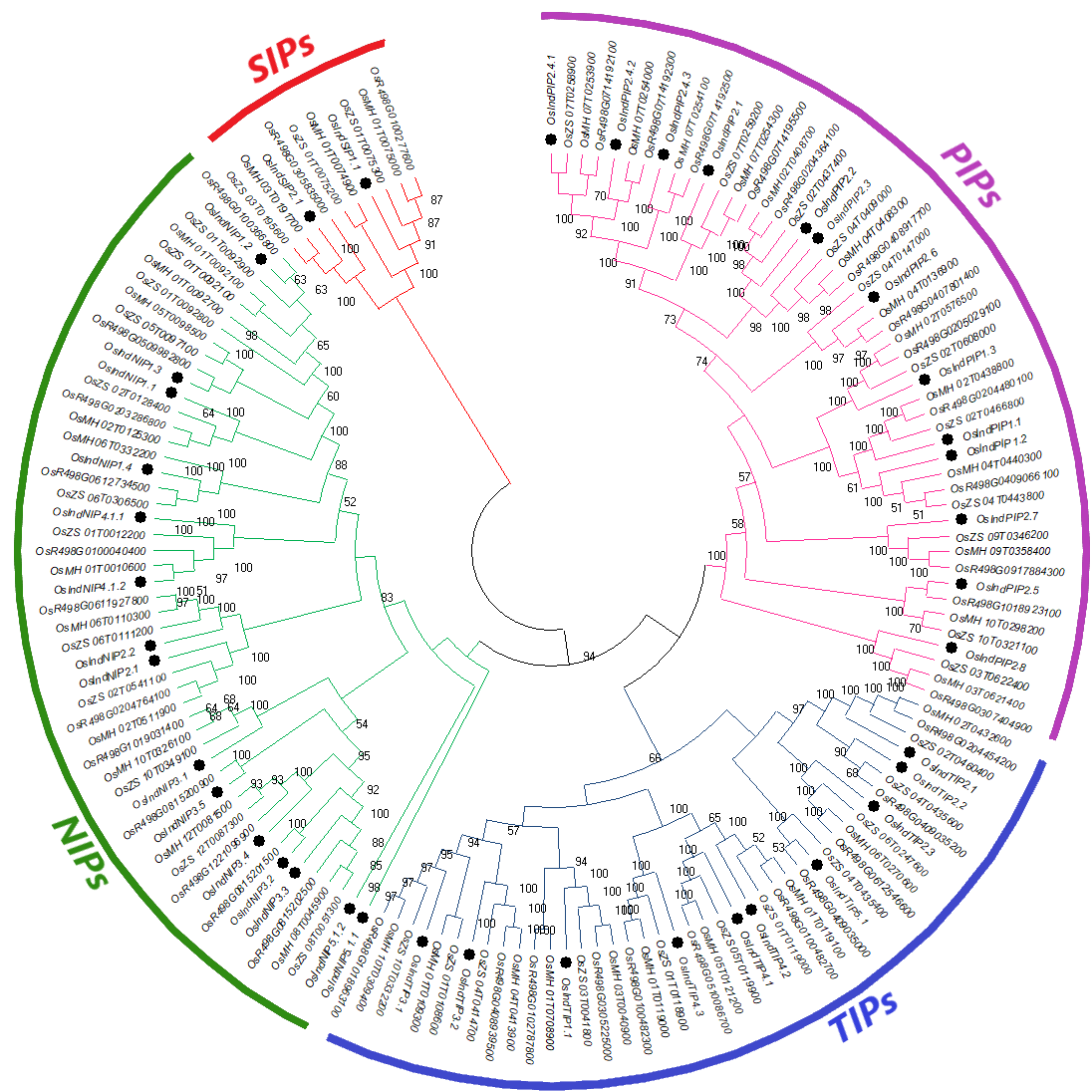


**Fig. S1A:** Comparative phylogenetic tree of four *indica* rice aquaporins. The aquaporin amino acid sequences of four important *indica* rice genotypes (93-11, Minghui 63, Shuhui498 and Zhenshan 97) were MAFFT aligned, and a Neighbor-Joining tree was inferred after computing evolutionary distances with Poisson correction method and complete deletion option in MEGA 7. The 93-11 aquaporins are indicated with solid black circles. Bootstrap values ≥50% as computed from 1000 replicates are shown at tree nodes.


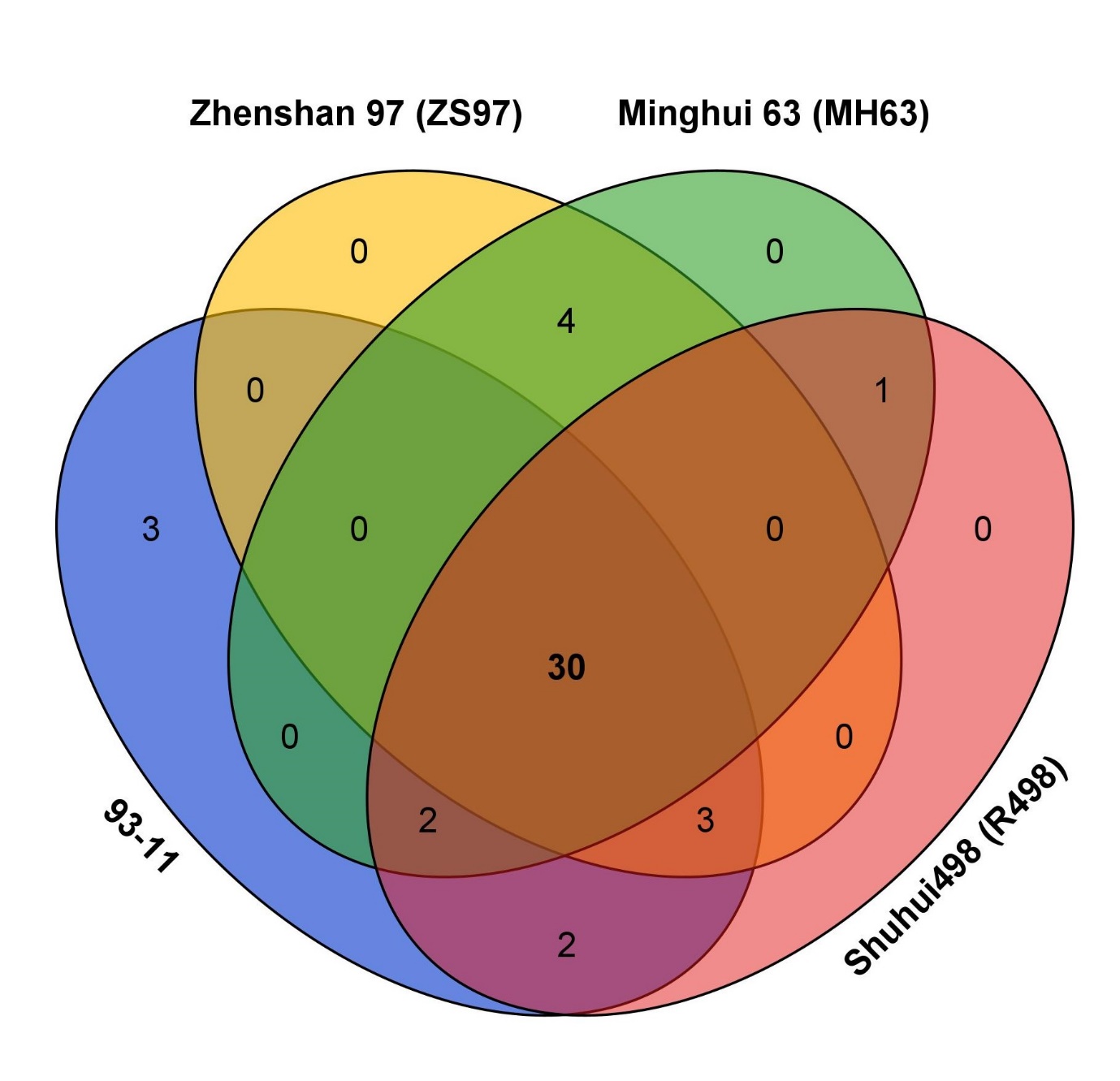


**Fig. S1B:** Comparison of number of aquaporins among four important *indica* rice genotypes.


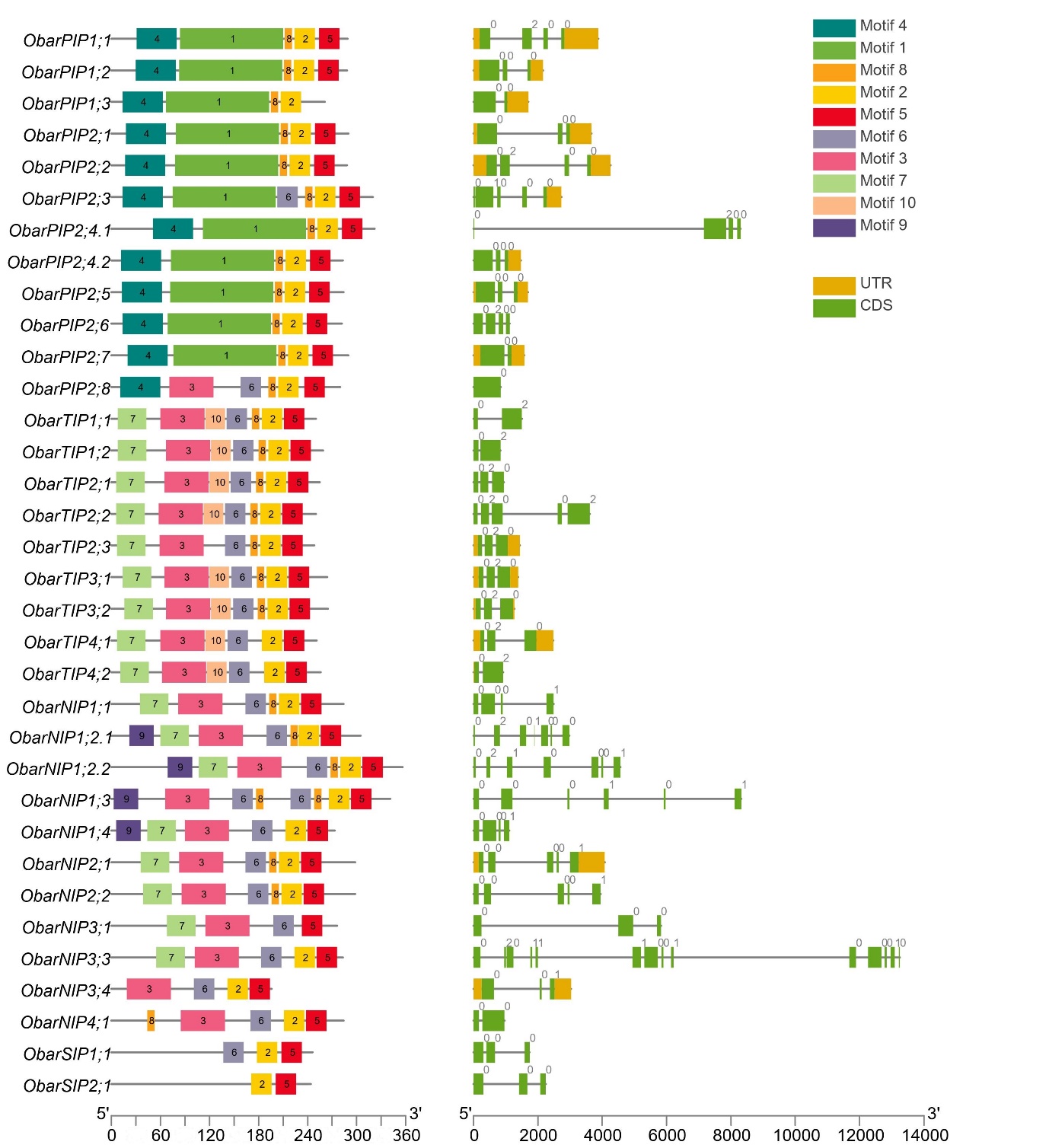


**Fig. S2:** Evolutionary conserved motifs and intron-exon distribution in *O. barthii* aquaporins.


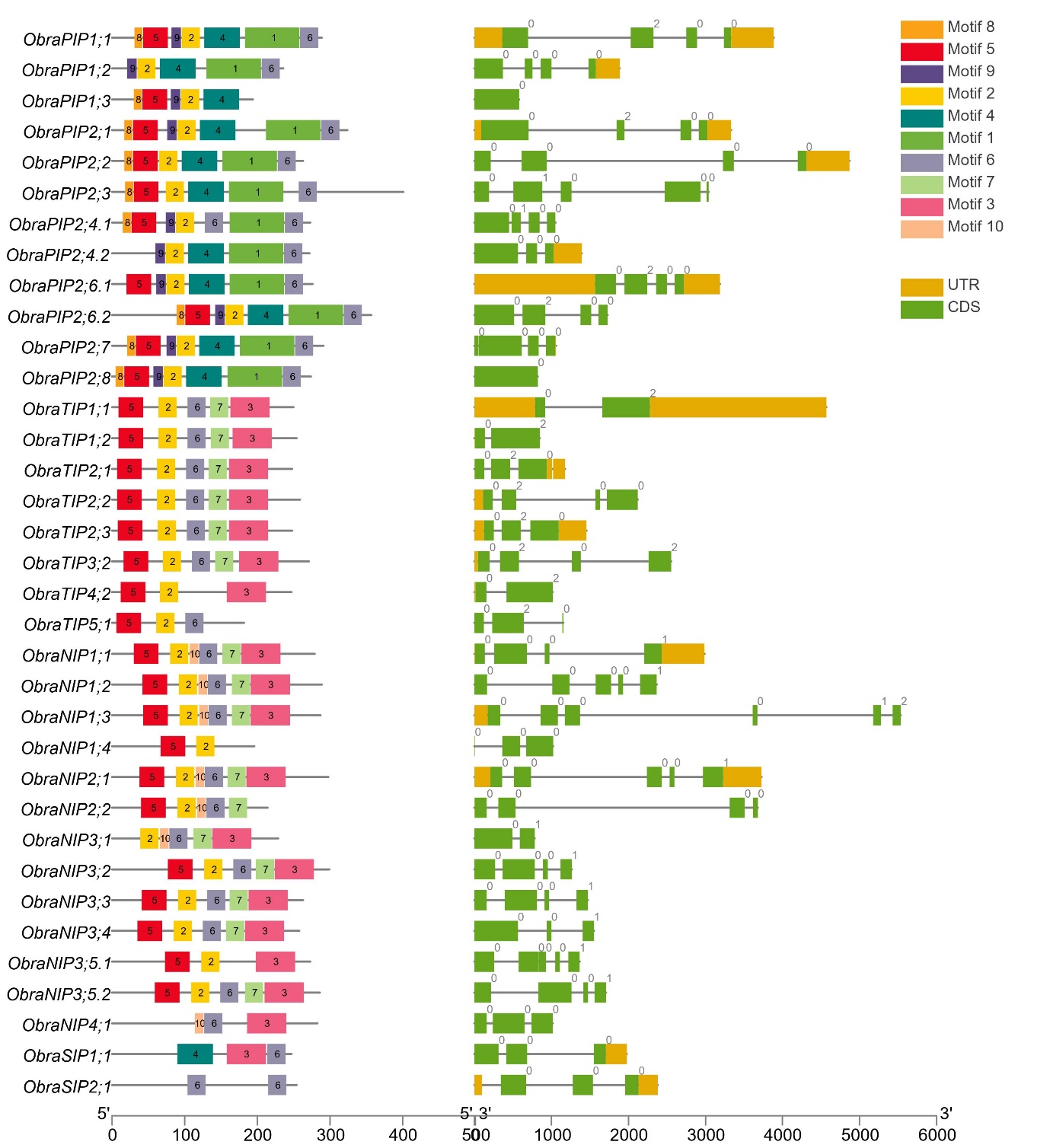


**Fig. S3:** Evolutionary conserved motifs and intron-exon distribution in *O. brachyantha* aquaporins.


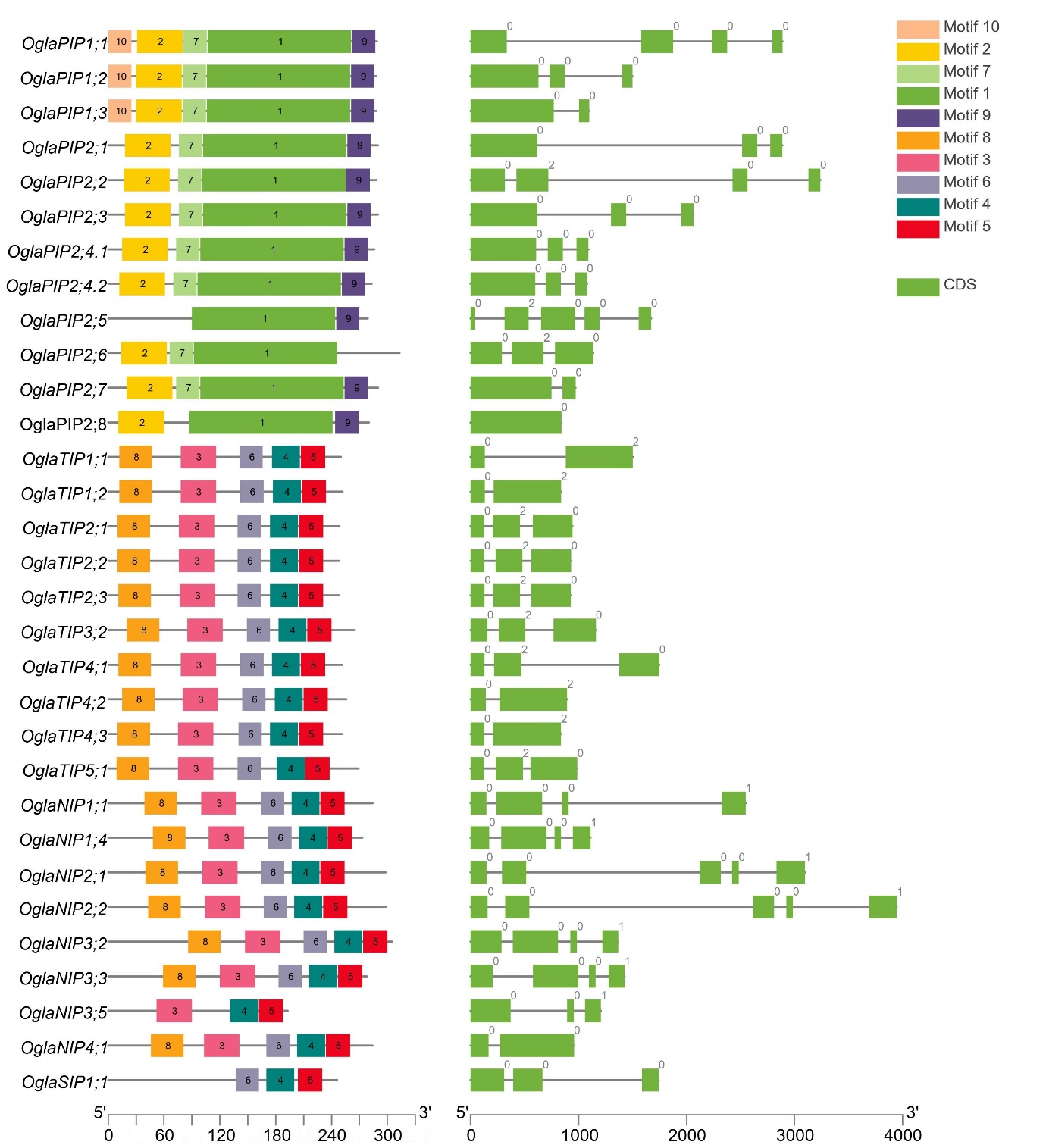


**Fig. S4:** Evolutionary conserved motifs and intron-exon distribution in *O. glaberrima* aquaporins.


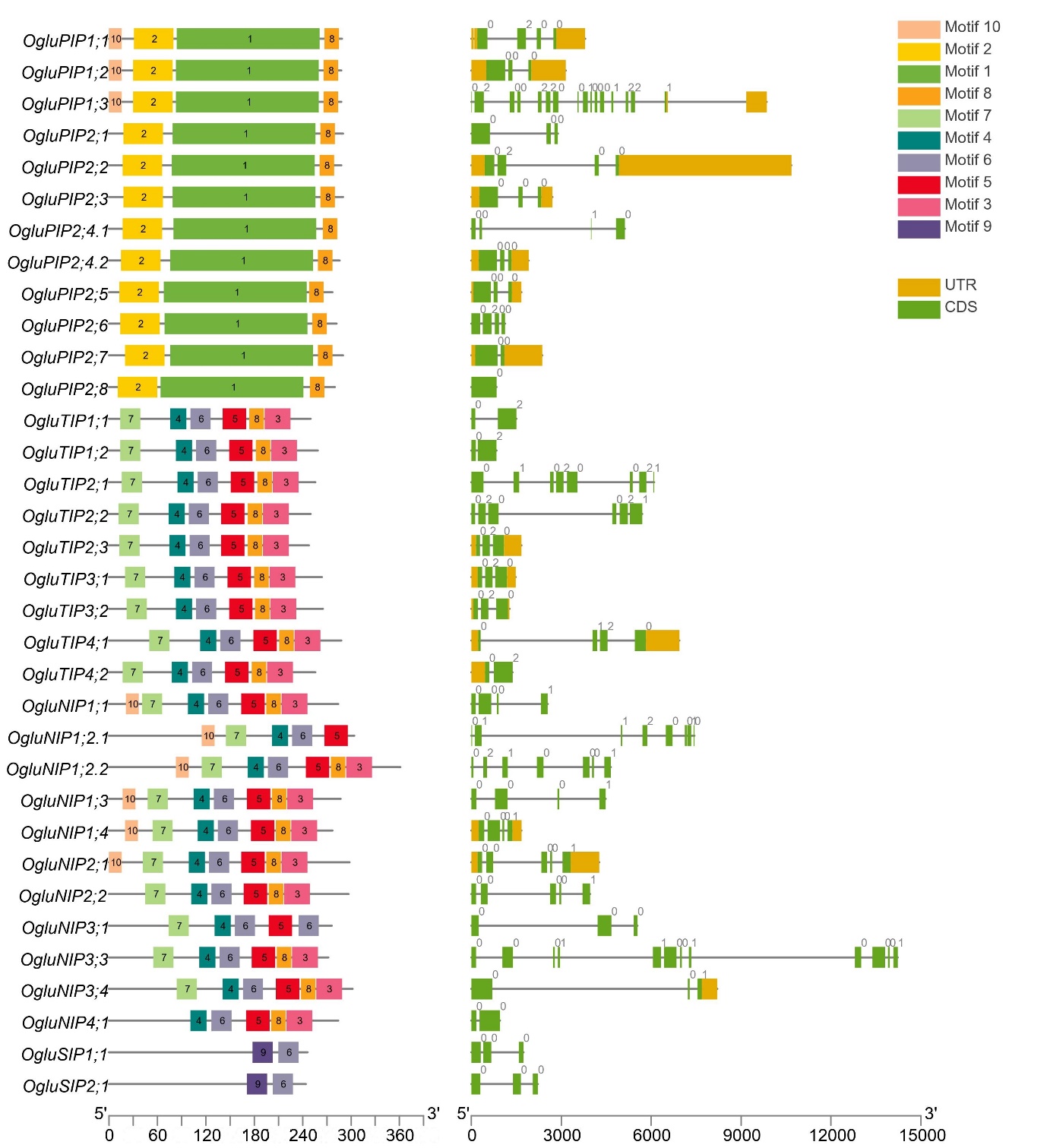


**Fig. S5:** Evolutionary conserved motifs and intron-exon distribution in *O. glumipatula* aquaporins.


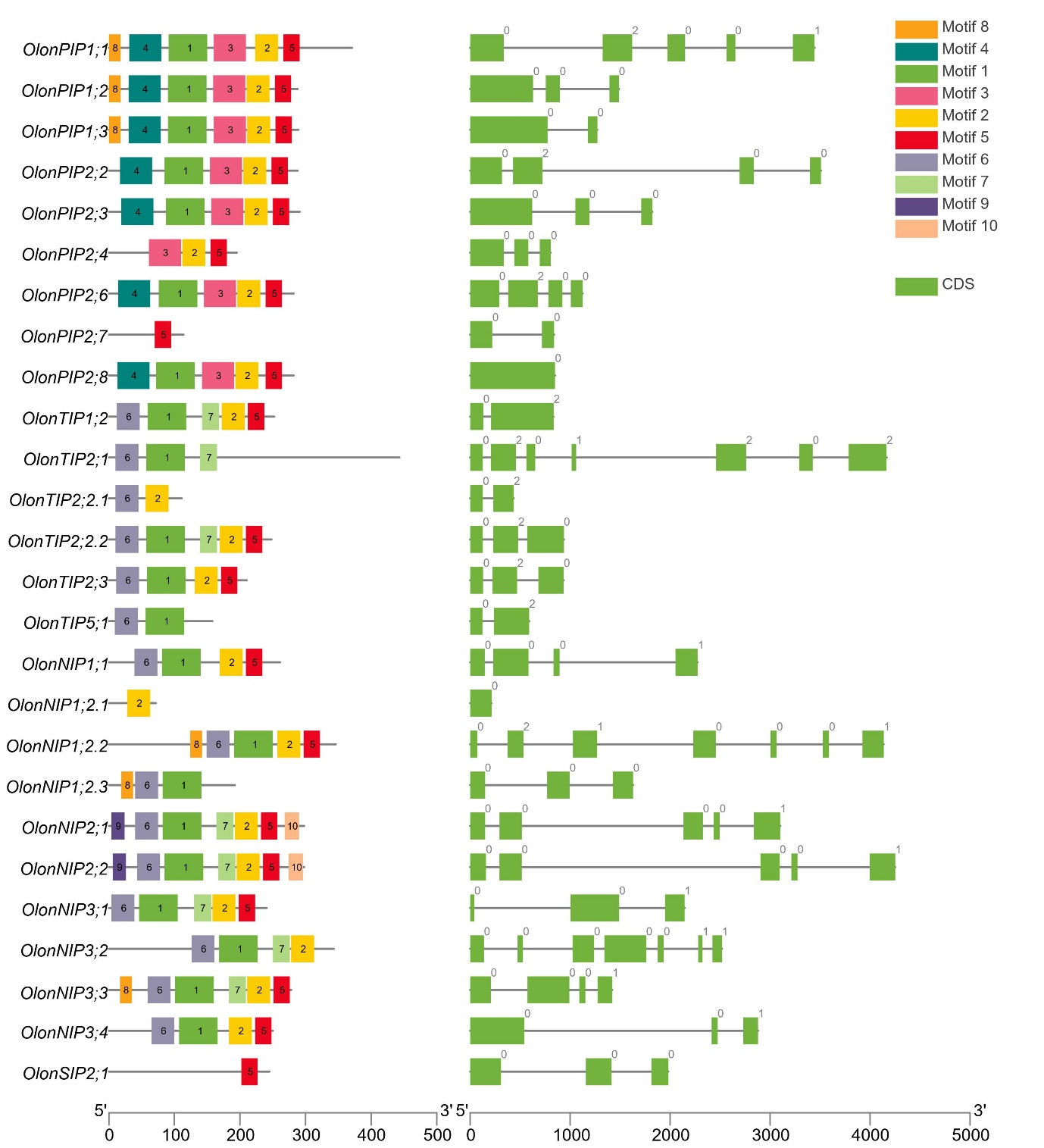


**Fig. S6:** Evolutionary conserved motifs and intron-exon distribution in *O. longistaminata* aquaporins.


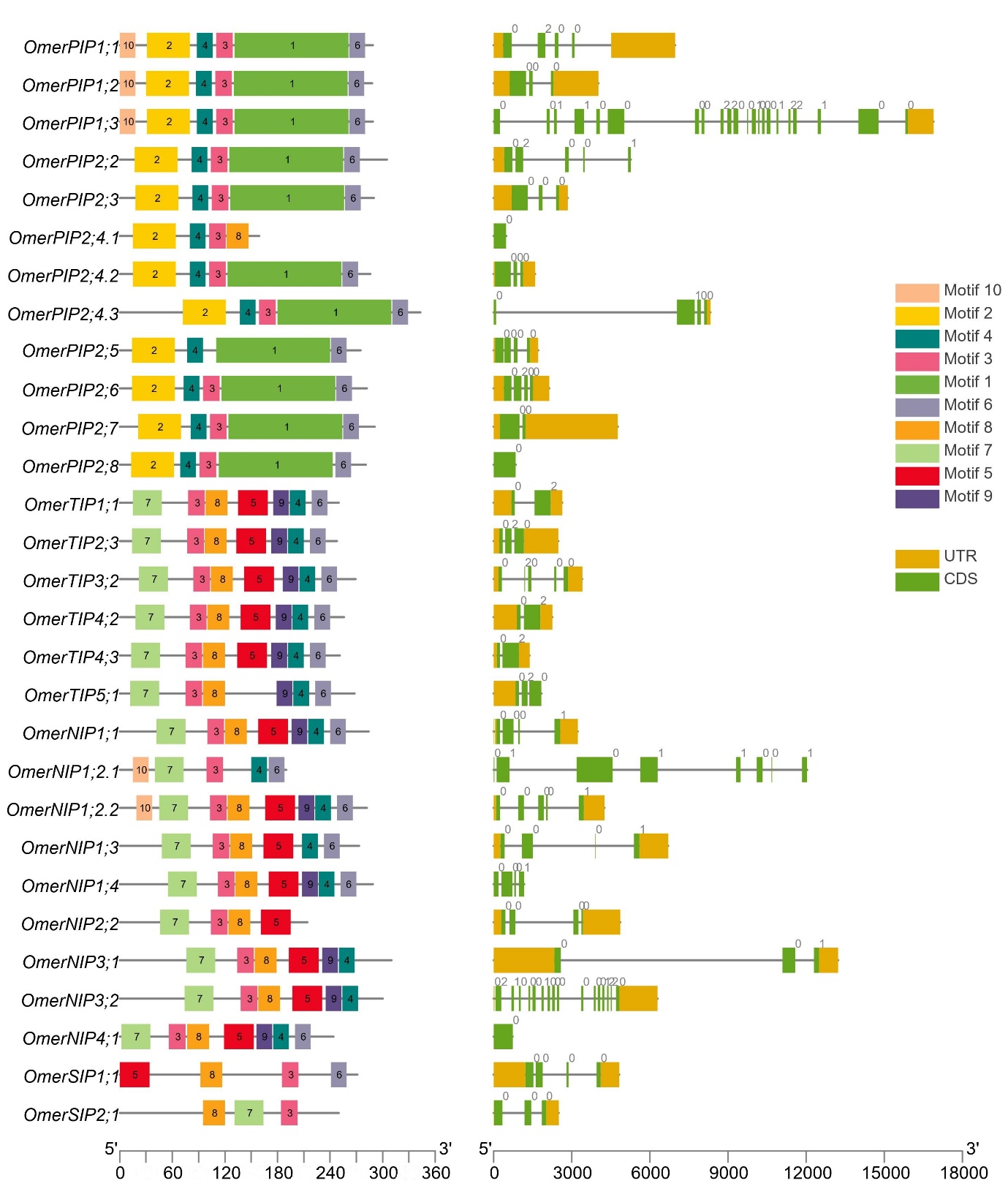


**Fig. S7:** Evolutionary conserved motifs and intron-exon distribution in *O. meridionalis* aquaporins.


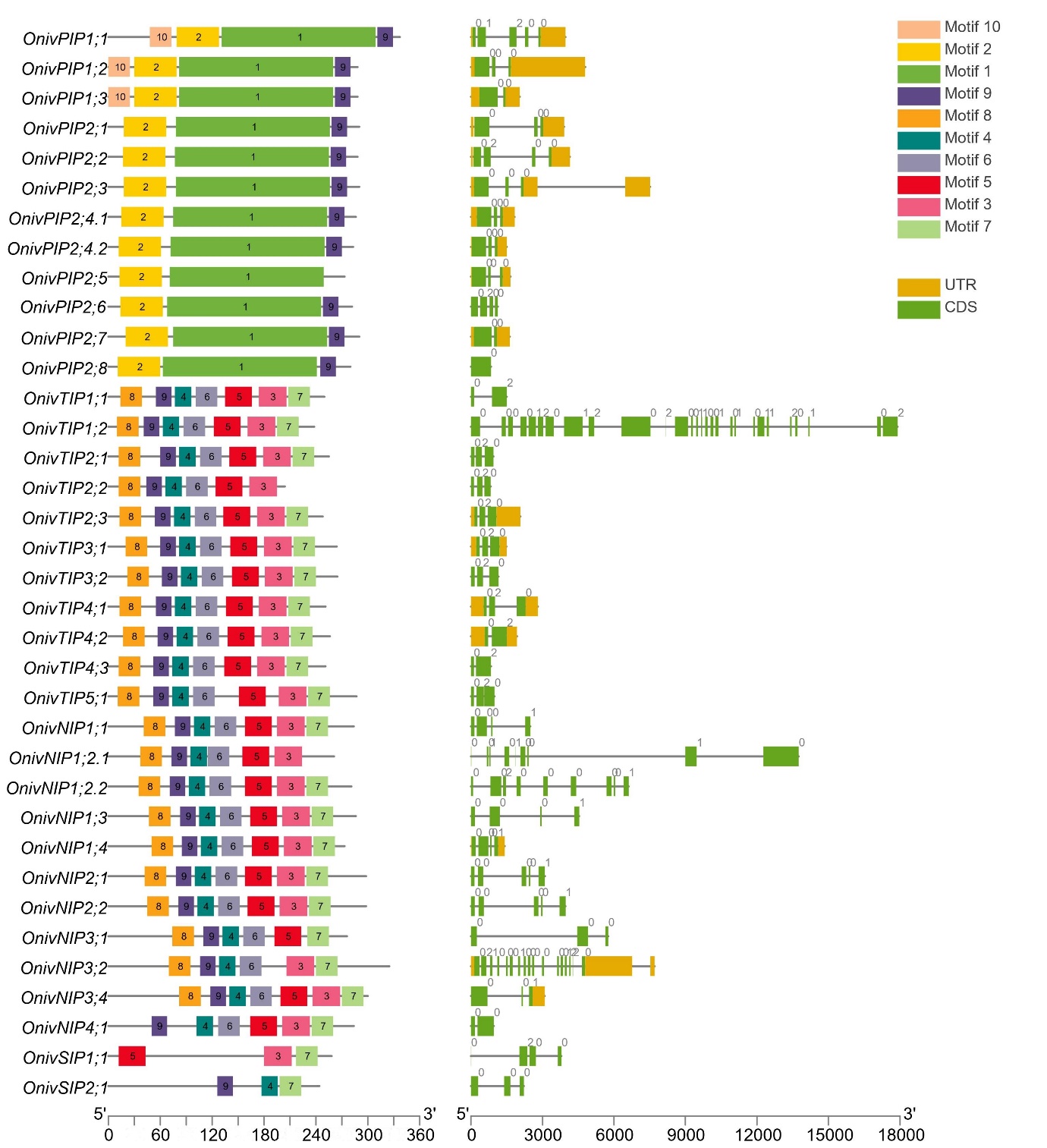


**Fig. S8:** Evolutionary conserved motifs and intron-exon distribution in *O. nivara* aquaporins.


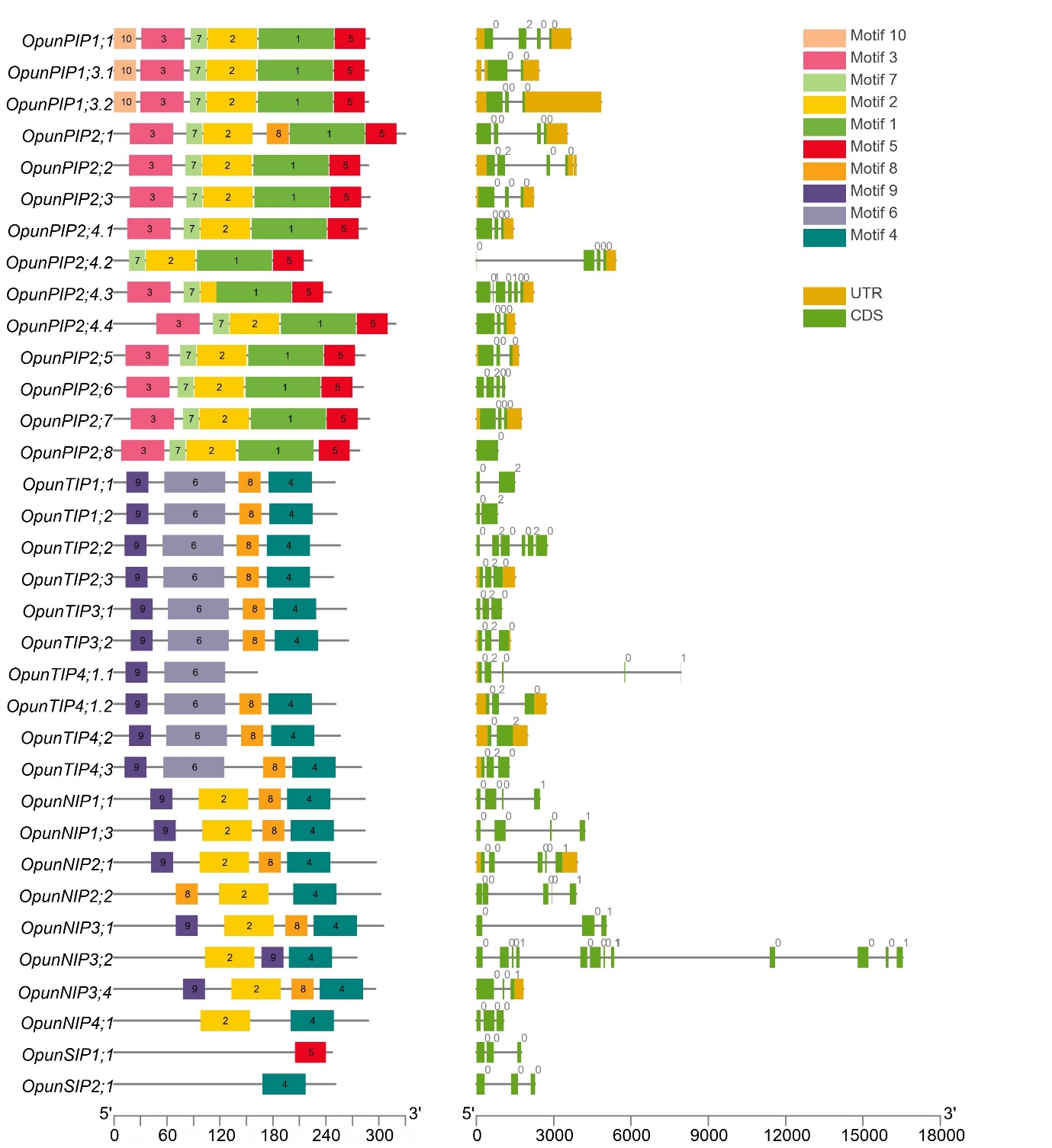


**Fig. S9:** Evolutionary conserved motifs and intron-exon distribution in *O. punctata* aquaporins.


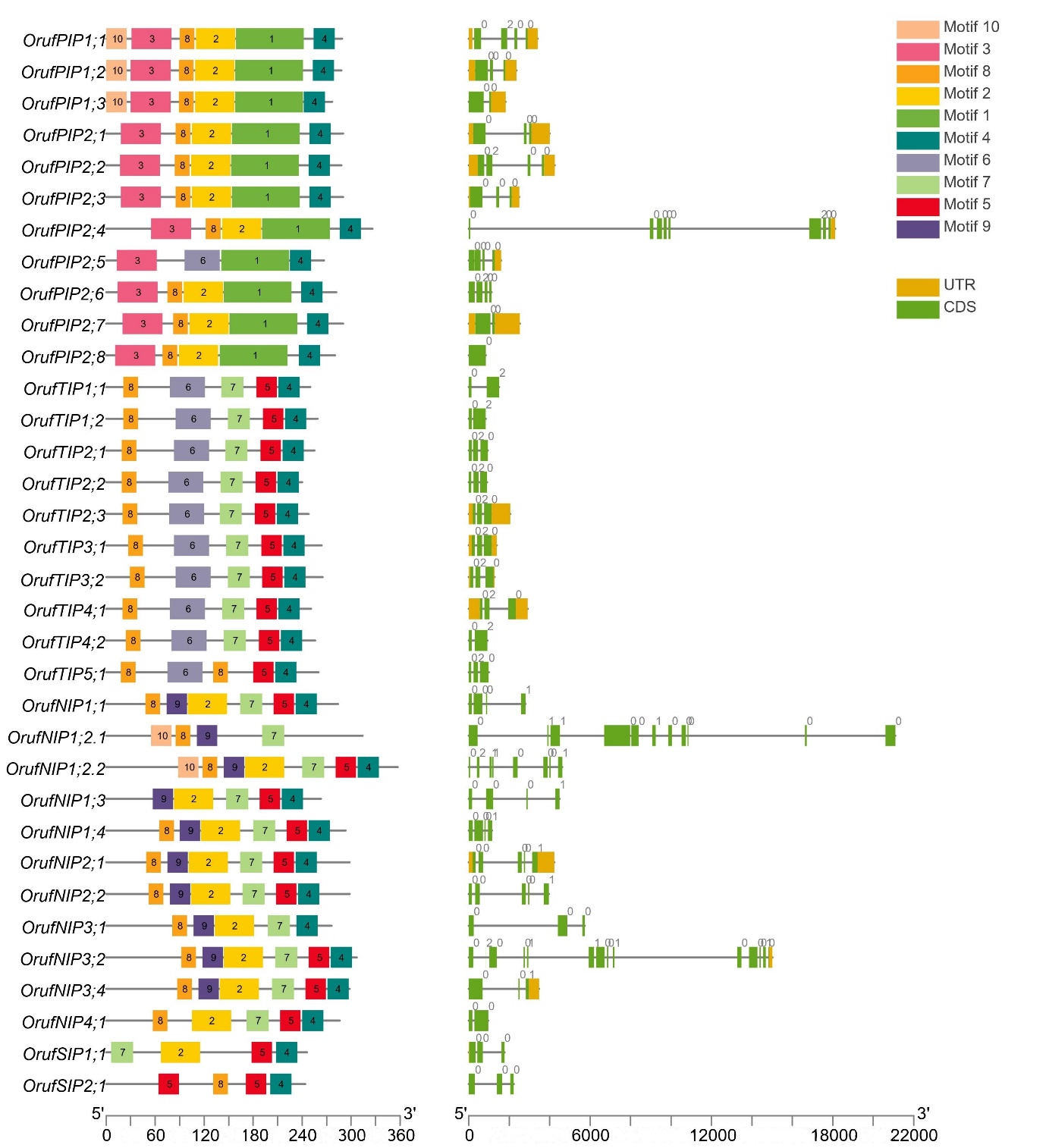


**Fig. S10:** Evolutionary conserved motifs and intron-exon distribution in *O. rufipogon* aquaporins.
